# Serum and CSF Metabolites in Stroke-Free Patients Are Associated With Vascular Risk Factors and Cognitive Performance

**DOI:** 10.3389/fnagi.2020.00193

**Published:** 2020-07-22

**Authors:** Sisi Peng, Ying Shen, Min Wang, Junjian Zhang

**Affiliations:** ^1^Department of Neurology, Zhongnan Hospital of Wuhan University, Wuhan, China; ^2^Department of Laboratory Medicine, Tongji Hospital, Tongji Medical College, Huazhong University of Science and Technology, Wuhan, China; ^3^Public Technological Service Center, Institute of Hydrobiology, Chinese Academy of Sciences, Wuhan, China

**Keywords:** vascular cognitive impairment, cerebrovascular risk factors, Framingham stroke risk profile, cerebrospinal fluid, metabolomics

## Abstract

**Background and purpose**: The aggregation of vascular risk factors (VRFs) can aggravate cognitive impairment in stroke-free patients. Metabolites in serum and cerebrospinal fluid (CSF) may irreversibly reflect early functional deterioration. This study evaluated small-molecule metabolites (<1,000 Da) in the serum and CSF of patients with different degrees of cerebrovascular burden and investigated the correlation between metabolism and cognitive performance associated with VRFs.

**Methods**: The subjects were divided into a low-risk group (10-year stroke risk ≤ 5%), a middle-risk group (10-year stroke risk >5% and <15%), and a high-risk group (10 years stroke risk ≥ 15%) according to the Framingham stroke risk profile (FSRP) score, which was used to quantify VRFs. We assess the cognitive function of the participants. We semiquantitatively quantified the small molecules using liquid chromatography–tandem mass spectrometry (LC-MS/MS). The correlation between the small molecules and cognitive function, along with VRFs, was investigated to identify key small molecules and possible underlying metabolic pathways.

**Results**: When the FSRP scores increased, the cognitive performances of the subjects decreased, specifically the performance regarding the tasks of immediate memory, delayed recall, and executive function. Seven metabolites (2-aminobutyric acid, Asp Asp Ser, Asp Thr Arg, Ile Cys Arg, 1-methyluric acid, 3-tert-butyladipic acid, and 5α-dihydrotestosterone glucuronide) in serum and three metabolites [Asp His, 13-HOTrE(r), and 2,5-di-tert-Butylhydroquinone] in CSF were significantly increased, and one metabolite (arachidonoyl PAF C-16) in serum was significantly decreased in high-risk group subjects. Among these metabolites, 1-methyluric acid, 3-tert-butyladipic, acid and Ile Cys Arg in serum and 13-HOTrE(r), 2,5-di-tert-butylhydroquinone, and Asp His in CSF were found to be negatively related with cognitive performance in the high-risk group. Arachidonoyl PAF C-16 in serum was found to be associated with better cognitive performance. Caffeine metabolism and the tricarboxylic acid cycle (TCA cycle) were identified as key pathways.

**Conclusions**: 1-Methyluric acid, 3-tert-butyladipic acid, arachidonoyl PAF C-16, and Ile Cys Arg in serum and 13-HOTrE(r), 2,5-di-tert-butylhydroquinone, and Asp His in CSF were identified as potential biomarkers of vascular cognitive impairment (VCI) at the early stage. Caffeine metabolism and the TCA cycle may play important roles in the pathophysiology of VRF-associated cognitive impairment.

## Introduction

With the prevalence of an ageing society, dementia has become a growing public health issue. Vascular dementia (VaD), the advanced stage of vascular cognitive impairment (VCI), is the second most common cause of dementia after Alzheimer’s disease (AD), accounting for approximately 15% of cases (O’Brien and Thomas, 2015). Mounting population-based data have suggested that vascular risk factors (VRFs), such as aging, hypertension, diabetes mellitus (DM), and cardiovascular disease (CVD), substantially increase the risk of VaD (Debette et al., 2011; Unverzagt et al., 2011) free from defined stroke or small cerebrovascular lesions and aggravate the pathologic progression of AD (Lo and Jagust, 2012; Arvanitakis et al., 2016; Gottesman et al., 2017). Thus, long-term VRFs might have a direct effect on cognitive impairment. A substantial proportion of cognitive decline cases might be attributable to potentially modifiable risk factors (Baumgart et al., 2015; Van der Flier et al., 2018). The Rotterdam study suggested that, if modifiable risk factors were eliminated, most dementia cases could be prevented (de Bruijn et al., 2015). It is of significant importance to focus on modifiable risk factors that are prevalent in middle-aged and elderly people, who have a higher quality-of-life expectancy in general (Ngandu et al., 2015; Lipnicki et al., 2019). Nevertheless, VCI, as a non-single-risk-factor disease, is inherently heterogeneous. Studying only one VRF associated with VCI is not sufficiently comprehensive and rigorous. The Framingham stroke risk profile (FSRP), used to estimate the 10-year risk for stroke based on prevalent VRFs, including age, hypertension, and CVD (D’Agostino et al., 1994), can evaluate multiple important risk factors simultaneously. Thus, the FSRP is a trustworthy tool to quantify the combination of VRF burden in stroke-free individuals (Unverzagt et al., 2011).

However, the principal pathophysiological mechanisms through which VRF burden leads to cognitive impairment are not fully understood. Evidence has suggested that many patients with VRFs have altered brain metabolism and even mild cognitive impairment before structural changes in the brain. A functional magnetic resonance study has shown that abnormal concentrations of glutamate and other brain metabolites may play an important role in the pathophysiology of VRF-associated cognitive impairment (Sun et al., 2014), which is the initial stage of VCI. Numerous studies have shown that VRFs, such as CVD, smoking, and DM, can disturb cerebral metabolism (Hsu et al., 2016; Yu et al., 2016; Ruiz-Canela et al., 2017). Previous scientific studies have also identified the associations between different subtypes of cognitive impairment and changes in serum metabolites (Kaddurah-Daouk et al., 2013; Mapstone et al., 2014; Wang et al., 2014) but have rarely investigated metabolites in cerebrospinal fluid (CSF). A more comprehensive view of the metabolism profile of VCI is crucial in clinical practice because it will help clinicians to identify individuals at risk of developing cognitive impairment at the early stage. Metabolomics is a new technology to quantify the metabolites of small molecules (<1,000 Da) in biological samples by a comprehensive metabolic profiling approach (Fiehn, 2002). Characterizations of the metabolic composition of serum and CSF are essential to understand the basic pathogenesis of VCI, gain knowledge about the pathophysiological process of VCI, and identify biomarkers for the early diagnosis or prognosis of VCI.

Here, we focus on middle-aged and elderly people with VRFs without dementia or stroke. We examined the association of the VRF burden estimated by the FSRP and cognitive impairment using conventional cognitive tests and generated a metabolism profile of small molecules in the serum and CSF samples of middle-aged and older adults in China. We found that a higher total FSRP score was positively related to significant VRF-associated cognitive decline. Four metabolites in serum and three metabolites in CSF were identified as potential biomarkers of VCI at the early stage. Additionally, two metabolic pathways may play an important role during this process.

## Materials and Methods

### Participants

This study was a cross-sectional study of patients aged ≥ 50 years who were hospitalized and examined at Zhongnan Hospital of Wuhan University. Other inclusion criteria included the following: (1) the patient showed no obvious vascular lesions by brain magnetic resonance imaging (MRI; Jianping, 2011; no vascular lesions in crucial sites, and the number of vascular lesions >1 cm in noncritical parts ≤ 3); (2) the patient did not meet the standard of dementia (McKhann et al., 1984); and (3) the patient signed informed consent.

**Figure 1:**
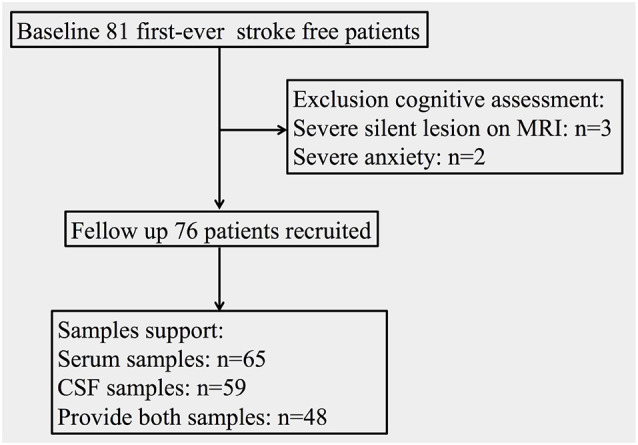
Flowchart of the population and samples support situation.

The exclusion criteria were as follows: (1) a clear history of stroke or neurological deficit localization; (2) severe respiratory, digestive, heart disease (rheumatic heart disease, valvular heart disease, cardiac stenting, and previous cardiac surgery), nervous system disease (Parkinson’s disease, epilepsy, and multiple sclerosis), or brain trauma history; (3) obesity, body mass index (BMI) ≥ 27, or other metabolic diseases; (4) any contraindications for MRI, such as claustrophobia and metal implants; (5) a family history of dementia or other causes of dementia; (6) severe anxiety, depression, recent life events, and inability to cooperate (aphasia, deafness); and (7) low education level (<5 years);

As shown in Figure 1, 76 stroke-free subjects (45 male and 31 female; mean age: 63.72 ± 8.00 years) with or without various VRFs were finally consecutively recruited in this study. Sixty-five of the subjects provided serum samples (41 male and 24 female; mean age: 64.49 ± 7.93 years), while 59 of the patients provided CSF samples (34 male and 25 female; mean age: 64.05 ± 7.68 years). This study was approved by the medical ethics committee of Zhongnan Hospital, Wuhan, China (clinical research registration number 2016007). Informed consent was obtained from all subjects and their nearest relatives.

### Clinical Characteristics Collection

We collected the following clinical characteristics from the subjects: age, sex, education level, hypertension, DM, CVD, atrial fibrillation (AF), current smoking status, left ventricular hypertrophy (LVH) on ECG, and systolic blood pressure (SBP). We also recorded some indicators that may potentially affect cognition: the concentrations of blood urea nitrogen (BUN), uric acid (UA; Ye et al., 2016), total cholesterol (TC), triglyceride (TG; Whitmer et al., 2005), high-density lipoprotein (HDL), low-density lipoprotein (LDL; Fanning et al., 2014), and apolipoprotein-A (APO-A) in blood.

SBP was based on the average of three measurements taken on the right arm with the informant in a seated position after 5 min of rest. DM was defined as a fasting glucose level greater than or equal to 7.0 mmol/L, a nonfasting glucose level ≥ 11.1 mmol/L, or the self-reported use of hypoglycemic agents or insulin. Current smoking status was divided into current cigarette smokers (regular smoking in the past 1 year) or noncurrent cigarette smokers. With self-reported CVD (myocardial infarction, angina pectoris, coronary insufficiency, intermittent claudication, or congestive heart failure), AF, and LVH on ECG (V1S+V5R > 4.0 mV for men or >3.5 mV for women), the disease history was diagnosed (Soliman et al., 2010).

### Framingham Stroke Risk Profile

The FSRP (Wolf et al., 1991; D’Agostino et al., 1994) was developed by the Framingham Heart Research Center in the United States to predict the probability of a 10-year stroke incidence in stroke-free middle-aged and elderly individuals. The FSRP is calculated based on the different weights of the VRFs. Common risk factors, such as age, SBP, DM, CVD, AF, current smoking status, and LVH on ECG, are included in the FSRP. There are separate scores for men and women, and the score ranges from 3% to 88% for men and 1% to 84% for women. The final score of FSRP represents the 10-year probability of stroke (see the Framingham Heart Study Website for specific scoring methods)^1^. To comprehensively consider the VRFs of the subjects, we used the FSRP to quantify them. All the patients were divided into three groups according to their 10-year probability of stroke: low-risk group (≤5%), medium-risk group ( >5% and <15%), and high-risk group (≥15%).

### Neuropsychological Assessment

The cognitive assessments of all participants were assessed by highly professional doctors. The Montreal Cognitive Assessment (MoCA; Nasreddine et al., 2005), Rey auditory verbal learning test (RAVLT), digital sign substitution test (DSST), digit span test (DST), and verbal fluency test (VFT) were applied to evaluate the cognitive function of the subjects. The five cognitive scales mentioned above were designed to estimate a full range of cognitive functions, including global cognitive function, memory (short-time memory, delayed memory), executive function, attention (also working memory), and verbal function, respectively. The Hamilton Anxiety Scale (HAMA; Hamilton, 1959) and the Hamilton Depression Scale (HAMD; Hamilton, 1960) were used to exclude those with obvious anxiety or clear depression.

### Sample Collection

Serum sample collection: the fasting whole-blood samples were collected at 8:00–10:00 AM, incubated at room temperature (25°C) for 30 min, and centrifuged at 3,500 rpm for 10 min. The supernatant was then transferred to a centrifuge tube. The supernatant was then centrifuged at 8,000 rpm for five additional minutes, and then 200 μl of the supernatant was transferred into cryopreservation tubes and stored at −80°C until measurement.

CSF sample collection: all CSF samples were collected during lumbar puncture before combined spinal anesthesia in patients undergoing elective surgery (implant removal of the low extremity and inguinal hernia). Once collected at 8:00–10:00 AM, the CSF was centrifuged immediately at 8,000 rpm for 10 min to discard possible cellular elements. After transfer of 200 μl of the supernatant into cryopreservation tubes, all CSF samples were stored at −80°C until further analysis.

### Sample Preparation

Serum sample processing steps: first, we mixed 140 μl of the serum sample with 420 μl of ice cold methanol, followed by vortexing the samples for 20 s. Thereafter, the samples were placed in 4°C environment for 1 h and were centrifuged at 14,600 r/min for 15 min. Finally, 350 μl of the supernatant was added to a PE tube, followed by drying at room temperature under N_2_. The dried sample was resuspended in 70 μl methanol–water (1:1, v/v), vortex-mixed for 20 s, and then centrifuged at 14,600 r/min for 10 min. The supernatant was stored for ultraperformance liquid chromatography–tandem mass spectrometry (UPLC-MS/MS) analysis. Quality control (QC) samples: 100 μl each of the processed serum samples was pooled after centrifugation at high speed, followed by mixing evenly. Next, we removed 350 μl of the pooled QC samples, followed by drying at room temperature under N_2_ and resuspension as described above for signal correction and quality assurance.

CSF sample processing steps: The same procedure was used as that for serum sample processing.

### Liquid Chromatography–Tandem Mass Spectrometry (LC-MS/MS)

LC-MS/MS analysis was performed using a UPLC Ultimate 3000 system coupled to a Q-Exactive mass spectrometer (Thermo Fisher Scientific, Bremen, Germany) and operated in the positive (ESI+) and negative (ESI−) electrospray ionization modes (one run for each mode). The system was controlled by Xcalibur 2.2 (Thermo Fisher Scientific). A HESI (heated electrospray ionization) source was used for both modes, a spray voltage of 3.8 kV was used for the positive mode, and 3.2 kV was used for the negative mode. The capillary temperature was 320°C, the sheath gas flow was 40 arbitrary units (AU), the auxiliary gas flow was 10 AU, the spare gas flow was 0 AU, and the S-lens RF level was 60 V. During the full-scan acquisition, which ranged from 70 to 1,000 m/z, the instrument was operated at 70,000 resolution (m/z = 200), with an automatic gain control (AGC) target of 3e6 charges and a maximum injection time (IT) of 200 ms. For MS2 analyses, the isolation window was set at 0.4 m/z, and the instrument was operated at 17,500 resolution (m/z = 200), with an AGC target of 1e5 charges, maximum IT of 50 ms, and stepped NCE of 20, 40, and 60 eV. The HRMS was coupled to an Ultimate WPS-3000 UHPLC chromatography system. The separation was carried out using an ACQUITY BEH C18 column (Waters, Ireland; 2.1 mm × 100 mm, 1.7 μm) stored at a temperature of 40°C. A multistep gradient (preceded by a 3-min equilibration time for 100% A) comprised mobile phase A consisting of 0.1% formic acid in water and mobile phase B consisting of methanol: 0–16 min, from 100% A to 100% B; 16–20 min, 100% B; 20–22 min, 100% A; the gradient operated at a flow rate of 0.3 ml/min over a run time of 22 min for both the negative and positive modes. The UHPLC autosampler temperature was set at 4°C, and the injection volume for each sample was 5 μl. The samples were randomized before the preanalytical step so that the injection order was independent of the clinical status. Four blanks and five QC samples were injected to equilibrate the system before each analytic series, and one QC sample was injected after every 10 samples to monitor the reproducibility of the liquid chromatography–high-resolution mass spectrum (LC-HRMS).

### UPLC-MS/MS Data Processing and Analysis

First, in the serum samples, 4,759 peaks were detected and 312 metabolites could be found using the interquartile range denoising method. However, in the CSF samples, 1,291 peaks were detected and 164 metabolites could be found. Next, the missing values of raw data were filled by half of the minimum value. Additionally, the total ion current normalization method was employed in this data analysis. The resulting three-dimensional data involving the peak number, sample name, and normalized peak area were input into the SIMCA14.1 software package (V14.1, MKS Data Analytics Solutions, Umea, Sweden) for principal component analysis (PCA) and orthogonal projections to latent structures–discriminate analysis (OPLS-DA). PCA showed the distribution of the origin data. To obtain a higher level of group separation and better understanding of the variables responsible for classification, supervised OPLS-DAs were applied. Sevenfold cross-validation was used to estimate the robustness and predictive ability of our model, and this permutation test was proceeded to further validate the model. The low values of the Q intercept indicate the robustness of the models, thus showing a low risk of overfitting and reliability. Based on the OPLS-DA, a loading plot was constructed, showing the contribution of variables to the difference between the groups. It also showed the important variables that were located far from the origin, but the loading plot is complex because of many variables. To refine this analysis, the first principal component of variable importance in the projection (VIP) was obtained. VIP values exceeding 1 were first selected as changed metabolites. In the next step, the remaining variables were assessed by student’s *t*-test (*P*-value < 0.1) and were discarded between two comparison groups. Additionally, commercial databases, including KEGG^2^ and MetaboAnalyst^3^, were utilized to identify the pathway analysis of metabolites.

### Quality Control Analysis

QC samples were analyzed to assess the reproducibility of the methods (Kamleh et al., 2012). The stability of mass accuracy and retention time were evaluated. Clustering of the QC samples was assessed by PCA according to the peak area data. The charts show the sample number from which the analytical system was equilibrated (Supplementary Figures S1, S2).

### Statistical Analyses

The clinical data were expressed as means ± standard error or number of cases (percentage). SPSS 22.0 (Chicago, IL, USA) was used for data analysis. Analysis of variance was used for continuous variables, while chi-squared analysis was used for categorical variables compared with the clinical and demographic characteristics in different groups. We used multivariate covariance analysis to compare the differences in the cognitive function between groups, using the degree of education as a covariate. Statistically significant correlations between metabolites and clinical data and between metabolites and cognition were determined by Spearman’s correlation analysis. Statistical significance was set at *P* < 0.05.

## Results

1. The demographic and clinical characteristics of all subgroups (patients who provided serum samples) were presented as means ± SD or number of cases (%) in Table 1. The mean age of all groups was 64.49 ± 7.93 years. The patients with a heavier VRF burden tended to be older (*P* < 0.01), and we observed a significant difference in the sex distribution, education years, hypertension, DM, CVD, AF, current smoking status, systolic blood pressure, levels of HDL and APO-A in serum, and FSRP score. Among all VRFs, hypertension (46.15%) accounted for the highest proportion. Women were less likely to experience VRFs than men, and we found fewer women in the high-risk group and more in the low-risk group than men. No significant difference was found in LVH on ECG and the levels of BUN, UA, TC, TG, and LDL in serum.

**Table 1:** Demographic and clinical characteristics of the subjects with different vascular risk factor (VRF) burdens who provided serum samples.

Characteristic	Low-risk group (*n* = 24)	Medium-risk group (*n* = 27)	High-risk group (*n* = 14)	F or *χ*^2^	*P*-value
Sex					
Male^a^ [N, (%)]	9 (13.85)	19 (29.23)	13 (20.00)	12.69	<0.01
Female^a^ [N, (%)]	15 (23.08)	8 (12.31)	1 (1.54)	12.69	<0.01
Age^a^ ($\bar{x}$ ± s, years)	58.54 ± 5.48	65.59 ± 6.51	72.57 ± 5.76	25.00	<0.01
Education ($\bar{x}$ ± s, years)	9.13 ± 2.88	7.93 ± 2.29	10.21 ± 3.02	3.55	<0.05
Hypertension^a^ [N, (%)]	4 (6.15)	16 (24.62)	10 (15.38)	13.86	<0.01
DM^a^ [N, (%)]	0 (0.00)	3 (4.62)	4 (6.15)	7.52	<0.05
CVD^a^ [N, (%)]	1 (1.54)	2 (3.08)	6 (9.23)	12.70	<0.01
AF^a^ [N, (%)]	0 (0.00)	0 (0.00)	4 (6.15)	15.53	<0.01
Current Smoking^a^ [N, (%)]	0 (0.00)	8 (12.31)	4 (6.15)	8.62	<0.05
LVH^a^ [N, (%)]	1 (1.54)	0 (0.00)	0 (0.00)	1.74	0.42
SBP^a^ (mmHg)	126.38 ± 9.22	138.41 ± 13.96	134.64 ± 13.33	6.24	<0.01
BUN ($\bar{x}$ ± s, mmol/L)	5.19 ± 1.35	5.71 ± 1.92	5.95 ± 1.44	1.23	0.33
UA ($\bar{x}$ ± s, μmol/L)	310.58 ± 72.61	344.00 ± 107.71	377.57 ± 72.01	2.59	0.08
TC ($\bar{x}$ ± s, mmol/L)	4.37 ± 0.86	4.38 ± 1.06	3.90 ± 0.88	1.39	0.26
TG ($\bar{x}$ ± s, mmol/L)	1.35 ± 0.73	1.51 ± 0.74	2.24 ± 3.57	1.19	0.31
HDL ($\bar{x}$ ± s, mmol/L)	1.41 ± 0.36	1.33 ± 0.44	1.01 ± 0.37	4.57	<0.05
LDL ($\bar{x}$ ± s, mmol/L)	2.44 ± 0.66	2.46 ± 0.78	2.13 ± 0.53	1.21	0.31
APO-A ($\bar{x}$ ± s, mmol/L)	1.56 ± 0.36	1.52 ± 0.34	1.19 ± 0.32	5.59	<0.01
FSRP ($\bar{x}$ ± s, %)	3.04 ± 1.30	8.70 ± 2.20	20.57 ± 4.40	200.22	<0.01

*Abbreviations: DM, diabetes mellitus; CVD, cardiovascular disease; AF, atrial fibrillation; LVH, left ventricular hypertrophy on ECG; SBP, systolic blood pressure; BUN, blood urea nitrogen; UA, uric acid; TC, total cholesterol; TG, triglyceride; HDL, high-density lipoprotein; LDL, low-density lipoprotein; APO-A, apolipoprotein-A; FSRP, Framingham stroke risk profile. Note: “a” indicates the risk factors for Framingham stroke risk profile. The data are presented as means ± SD or number of cases (%)*.

2. As the vascular burden increased, we observed a significant decrease in the subjects’ whole-brain cognitive function (*p* < 0.01, assessed by the MoCA test), memory function, and executive function (*p* < 0.01, assessed by DSST). Regarding memory function, both immediate memory (*p* < 0.01, assessed by RAVLT) and delayed memory function (*p* < 0.05, assessed by RAVLT) were damaged. No significant difference was found in verbal fluency (*p* = 0.24, VFT), attention (*p* = 0.96, DST), anxiety (*p* = 0.90, assessed by HAMA), and depression (*p* = 0.68, assessed by HAMD; Table 2).

**Table 2:** Differences in cognitive performance in subgroups of subjects who provided serum samples.

	Low-risk group (*n* = 24)	Medium-risk group (*n* = 27)	High-risk group (*n* = 14)	*F*-value	*P*-value
MoCA	23.08 ± 2.73	21.11 ± 3.13	20.36 ± 3.52	4.71	<0.01
RAVLT (immediate memory)	35.92 ± 7.94	29.22 ± 8.36	29.21 ± 10.39	5.08	<0.01
RAVLT (delayed memory)	6.75 ± 1.98	4.93 ± 2.57	4.21 ± 3.24	4.28	<0.05
DST	11.25 ± 1.82	11.30 ± 2.05	11.07 ± 1.44	0.10	0.96
VFT	40.83 ± 6.18	37.48 ± 7.38	37.57 ± 5.91	1.44	0.24
DSST	29.29 ± 7.26	24.81 ± 7.49	23.93 ± 5.93	4.93	<0.01
HAMD	2.00 ± 1.91	1.52 ± 1.67	1.50 ± 1.35	0.51	0.68
HAMA	2.50 ± 1.62	2.67 ± 1.75	2.50 ± 1.87	0.20	0.90

*Abbreviations: MoCA, Montreal Cognitive Assessment; RAVLT, Rey auditory verbal learning test; DST, digit span test; VFT, verbal fluency test; DSST, digital sign substitution test; HAMD, Hamilton Depression Scale; HAMA, Hamilton Anxiety Scale. The data are expressed as the means ± SD*.

3. Table 3 shows the demographic and clinical data of subjects (mean age: 64.54 ± 7.86 years) who provided cerebrospinal fluid samples. Women were less likely to experience VRFs than men, and a heavier VRF burden was linked to old age. Additionally, we found a significant difference in sex distribution, age, education years, hypertension, CVD, AF, current smoking status, SBP, BUN level, UA level, and FSRP score compared with those in all the subgroups. No significant difference was found in DM and LVH on ECG and the levels of TC, TG, LDL, HDL, and APO-A in blood.

**Table 3:** Demographic and clinical characteristics of the subjects with different VRF burdens who provided cerebrospinal fluid (CSF) samples.

Characteristic	Low-risk group (*n* = 26)	Medium-risk group (*n* = 22)	High-risk group (*n* = 11)	F or *χ*^2^	*P*-value
Sex					
Male^a^ [N, (%)]	8 (13.56)	15 (25.42)	11 (18.64)	16.77	<0.01
Female^a^ [N, (%)]	18 (30.51)	7 (11.86)	0 (0.00)	16.77	<0.01
Age^a^ ($\bar{x}$ ± s, years)	58.73 ± 6.01	66.55 ± 6.05	71.64 ± 4.93	22.04	<0.01
Education ($\bar{x}$ ± s, years)	8.54 ± 2.44	7.55 ± 2.22	10.00 ± 2.90	3.72	<0.05
Hypertension^a^ [N, (%)]	4 (6.78)	14 (23.73)	7 (11.86)	13.87	<0.01
DM^a^ [N, (%)]	0 (0.00)	2 (3.39)	1 (1.69)	2.49	0.29
CVD^a^ [N, (%)]	1 (1.69)	1 (1.69)	4 (6.78)	10.16	<0.01
AF^a^ [N, (%)]	0 (0.00)	0 (0.00)	3 (5.08)	13.79	<0.01
Current Smoking^a^ [N, (%)]	0 (0.00)	6 (10.17)	4 (6.78)	9.92	<0.01
LVH^a^ [N, (%)]	1 (1.69)	0 (0.00)	0 (0.00)	1.29	0.52
SBP^a^ (mmHg)	128.31 ± 8.75	137.55 ± 11.43	134.73 ± 10.95	5.07	<0.01
BUN ($\bar{x}$ ± s, mmol/L)	4.81 ± 1.42	5.49 ± 1.16	6.27 ± 1.20	5.25	<0.01
UA ($\bar{x}$ ± s, μmol/L)	303.46 ± 68.15	335.45 ± 101.28	395.82 ± 69.88	4.88	<0.05
TC ($\bar{x}$ ± s, mmol/L)	4.34 ± 0.86	4.47 ± 1.04	3.83 ± 0.82	1.76	0.18
TG ($\bar{x}$ ± s, mmol/L)	1.24 ± 0.63	1.49 ± 0.75	1.16 ± 0.51	1.18	0.32
HDL ($\bar{x}$ ± s, mmol/L)	1.50 ± 0.43	1.34 ± 0.46	1.13 ± 0.49	2.76	0.07
LDL ($\bar{x}$ ± s, mmol/L)	2.36 ± 0.66	2.42 ± 0.77	2.12 ± 0.55	0.70	0.50
APO-A ($\bar{x}$ ± s, mmol/L)	1.60 ± 0.38	1.53 ± 0.34	1.32 ± 0.48	2.04	0.14
FSRP ($\bar{x}$ ± s, %)	3.12 ± 1.21	8.86 ± 2.23	19.09 ± 3.91	189.09	<0.01

*Abbreviations: DM, diabetes mellitus; CVD, cardiovascular disease; AF, atrial fibrillation; LVH, left ventricular hypertrophy on ECG; SBP, systolic blood pressure; BUN, blood urea nitrogen; UA, uric acid; TC, total cholesterol; TG, triglyceride; HDL, high-density lipoprotein; LDL, low-density lipoprotein; APO-A, apolipoprotein-A; FSRP, Framingham stroke risk profile. Note: “a” indicates the risk factors for the Framingham stroke risk profile. The date are presented as means ± SD or number of cases (%)*.

4. Table 4 presents the significant difference of cognitive function in subjects who provided CSF samples. Regarding the VRFs, we observed that the subjects’ whole-brain cognitive function (*p* < 0.01, assessed by the MoCA test), immediate memory (*p* < 0.01, assessed by RAVLT), delayed memory function (*p* < 0.01, assessed by RAVLT), executive function (*p* < 0.01, assessed by DSST), and verbal fluency (*p* < 0.01, assessed by VFT) were all reduced. No differences were found in anxiety (*p* = 0.98, assessed by HAMA), depression (*p* = 0.88, assessed by HAMD), and working memory (*p* = 0.5, DST).

**Table 4:** Differences in cognitive performance in subgroups of subjects who provided CSF samples.

	Low-risk group (*n* = 26)	Medium-risk group (*n* = 22)	High-risk group (*n* = 11)	*F*-value	*P*-value
MoCA test	23.38 ± 2.28	21.05 ± 3.03	20.09 ± 3.75	7.32	<0.01
RAVLT (immediate memory)	34.85 ± 6.73	28.68 ± 6.90	26.55 ± 10.69	4.34	<0.01
RAVLT (delayed memory)	6.65 ± 1.57	4.68 ± 1.46	4.09 ± 3.33	6.03	<0.01
DST	11.73 ± 1.95	11.23 ± 1.69	11.09 ± 1.58	0.81	0.50
VFT	40.15 ± 5.71	36.41 ± 5.19	34.73 ± 3.13	4.21	<0.01
DSST	30.12 ± 7.10	24.73 ± 7.80	22.55 ± 5.63	4.51	<0.01
HAMD	1.69 ± 1.67	1.36 ± 1.59	1.36 ± 0.81	0.22	0.88
HAMA	2.38 ± 1.58	2.55 ± 1.57	2.36 ± 1.75	0.06	0.98

*Abbreviations: MoCA, Montreal Cognitive Assessment; RAVLT, Rey auditory verbal learning test; DST, digit span test; VFT, verbal fluency test; DSST, digital sign substitution test; HAMD, Hamilton Depression Scale; HAMA, Hamilton Anxiety Scale. The data are expressed as the means ± SD*.

### Metabolic Changes in Serum

**Figure 2:**
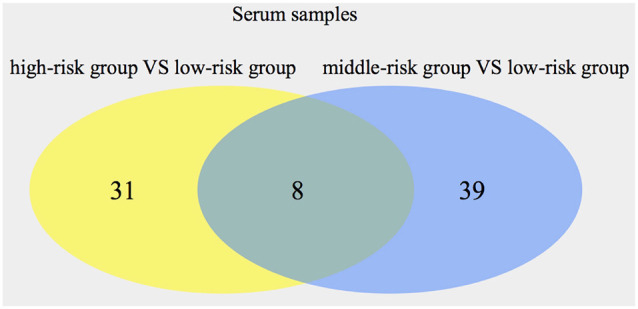
Numbers of significant metabolites comparing the high-risk group with the low-risk group, as well as the middle-risk group with the low-risk group, in serum. The intersection of the metabolites is also presented.

The OPLS-DA model was used, and the results are shown in the scatter plots of the OPLS-DA model scores (Supplementary Figures S3, S4). Comparing the high-risk group with the low-risk group, 39 metabolites (20 from POS, 19 from NEG) were obtained through multivariate analysis. When the middle-risk group was compared with the low-risk group, 47 metabolites (25 from POS, 22 from NEG) were found. Figure 2 shows the Venn diagram of all the metabolites showing significant differences in serum. Next, we selected 8 common metabolites and described them in detail in Table 5. 2-Aminobutyric acid, Asp Asp Ser, Asp Thr Arg, Ile Cys Arg, 1-methyluric acid, 3-tert-Butyladipic acid, and 5α-Dihydrotestosterone glucuronide exhibit increased levels in the higher risk group compared with that in the low-risk group. Conversely, arachidonoyl PAF C-16 presents a decreased level in the higher risk group.

**Table 5:** Eight common significantly changed metabolites in serum.

Metabolite	Ion mode	Regulated mode	rt	mz	MS^2^ score	VIP
2-Aminobutyric acid	POS	Up	106.08	11.72	0.77	1.90
Arachidonoyl PAF C-16	POS	Down	768.54	18.06	0.83	1.70
Asp Asp Ser	POS	Up	336.16	10.43	0.93	1.33
Asp Thr Arg	POS	Up	390.22	11.29	0.99	1.68
Ile Cys Arg	POS	Up	224.10	11.21	0.95	1.11
1-Methyluric acid	NEG	Up	181.06	3.41	0.90	1.86
3-tert-Butyladipic acid	NEG	Up	202.12	11.77	0.85	1.93
5α-Dihydrotestosterone glucuronide	NEG	Up	466.26	14.43	0.86	1.12

### Metabolic Changes in CSF

**Figure 3:**
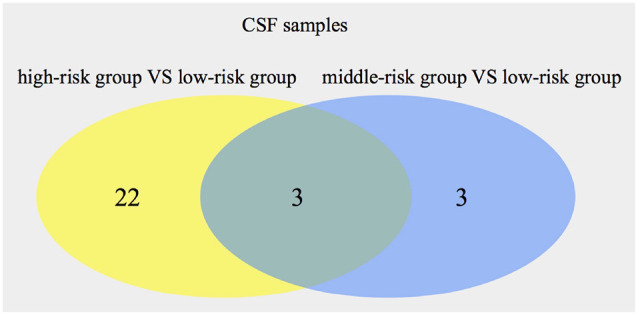
Numbers of significant metabolites comparing the high-risk group with the low-risk group, as well as the middle-risk group with the low-risk group, in cerebrospinal fluid (CSF). The intersection of the metabolites is also presented.

Using the same method, 25 metabolites (16 from POS, nine from NEG) were found by comparing the high-risk group with the low-risk group in CSF samples. Comparing the middle-risk group vs. the low-risk group, six metabolites (two from POS, four from NEG) were expressed (Supplementary Figures S5, S6). Figure 3 shows three common metabolites [Asp His, 13-HOTrE(r), 2,5-di-tert-Butylhydroquinone, described in Table 6] consistently enriched in groups with higher risk.

**Table 6:** Three common significantly changed metabolites in CSF.

Metabolite	Ion mode	Regulated mode	rt	mz	MS^2^ score	VIP
Asp His	POS	Up	266.11	12.23	0.93	1.32
13-HOTrE(r)	NEG	Up	11.64	294.18	0.98	3.12
2,5-di-tert-Butylhydroquinone	NEG	Up	11.64	222.16	0.87	3.11

### Correlation Between the Potential Metabolites and Cognitive Impairment

**Figure 4:**
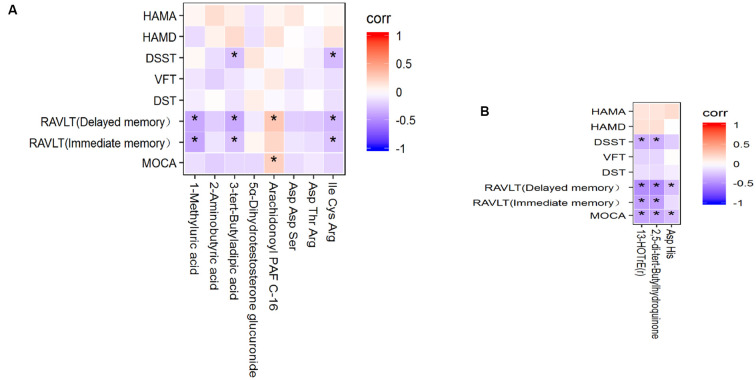
**(A)** Heatmap of the correlations between the 8 potential metabolites in serum and cognitive performance. **(B)** Heatmap of the correlations between the 3 potential metabolites in CSF and cognitive performance. *P < 0.05.

To evaluate the correlation between the common metabolites and cognitive impairment, Spearman’s correlation analysis was implemented. First, the correlation between serum metabolites and cognitive performance is shown in Figure 4A. 1-Methyluric acid showed a strong negative correlation with RVALT (delayed memory; corr = −0.372, *p* < 0.01) and RVALT (immediate memory; corr = −0.383, *p* < 0.01); 3-tert-butyladipic acid was significantly negatively correlated with RVALT (delayed memory; corr = −0.365, *p* < 0.01), RVALT (immediate memory; corr = −0.263, *p* < 0.05), and DSST (corr = −0.270, *p* < 0.05); arachidonoyl PAF C-16 was positively correlated with MoCA (corr = 0.250, *p* < 0.05) and RVALT (delayed memory; corr = 0.291, *p* < 0.05); Ile Cys Arg was negatively correlated with RVALT (delayed memory; corr = −0.302, *p* < 0.05), RVALT (immediate memory; corr = −0.256, *p* < 0.05), and DSST (corr = −0.293, *p* < 0.05). Second, the correlations between the CSF metabolites and cognitive performance are depicted in Figure 4B: 13-HOTrE(r) and 2,5-di-tert-butylhydroquinone were significantly negatively correlated with MoCA (corr = −0.391, *p* < 0.01; corr = −0.383, *p* < 0.01), RVALT (immediate memory; corr = −0.413, *p* < 0.01; corr = −0.414, *p* < 0.01), RVALT (delayed memory; corr = −0.490, *p* < 0.01; corr = −0.503, *p* < 0.01), and DSST (corr = −0.351, *p* < 0.01; corr = −0.346, *p* < 0.01); Asp His was negatively correlated with MoCA (corr = −0.281, *p* < 0.05) and RVALT (delayed memory; corr = −0.277, *p* < 0.05).

### Pathway Analysis and Possible Underlying Pathways

**Figure 5:**
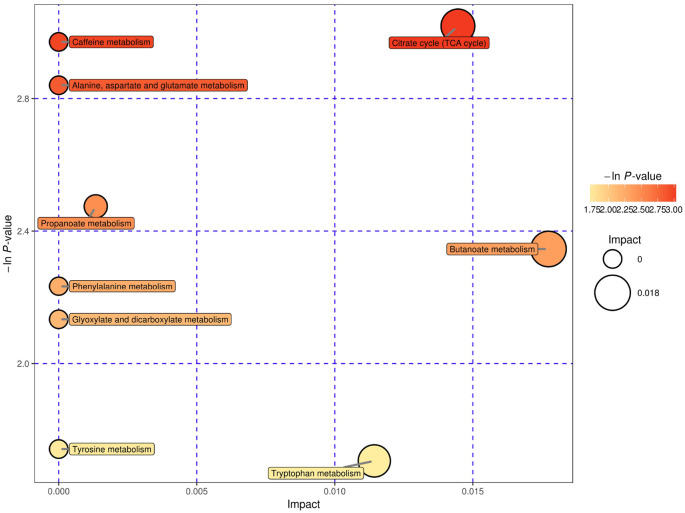
Serum, negative electrospray ionization (ESI−), and pathway analysis for the high-risk group vs. the low-risk group expressed as bubble plots. Significantly changed pathways based on the enrichment and topology analysis are shown. The x-axis represents pathway enrichment, and the y-axis represents the pathway impact. Large sizes and dark colors represent the major pathway enrichment and high pathway impact values, respectively.

**Figure 6:**
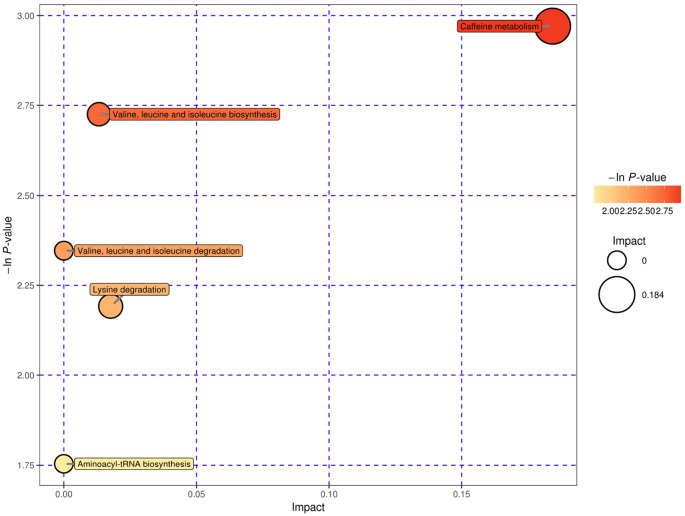
CSF, ESI+, and pathway analysis for the high-risk group vs. the low-risk group expressed as bubble plots. Significantly changed pathways based on the enrichment and topology analysis are shown. The x-axis represents pathway enrichment, and the y-axis represents the pathway impact. Large sizes and dark colors represent the major pathway enrichment and high pathway impact values, respectively.

We used bubble plots to explore meaningful differential metabolic pathways. Regarding the metabolites showing different levels in serum, we found that the caffeine metabolic pathway [impact = 0, −In (P) = 2.971] and citrate cycle (tricarboxylic acid cycle, TCA cycle) metabolic pathway [impact = 0.014, −In (P) = 3.018] were prominent when we compared the high-risk group with the low-risk group (Figure 5). The serum differential metabolite 1-methyluric acid mentioned above is involved in the caffeine metabolic pathway (Supplementary Figure S7). Regarding the CSF differential metabolites, the caffeine metabolic pathway [impact = 0.184, −In (P) = 2.971] calls our attention (Figure 6). It should be mentioned that, although caffeine is not the common metabolite consistently identified in groups with a higher risk both in serum and CSF samples, caffeine was present in the high-risk group compared with that in the low-risk group in CSF (ESI+).

## Discussion

In this study, we observed that a high FSRP score was positively associated with worsened cognitive impairment, and the cognitive domains that were damaged included immediate memory, delayed recall, and executive function. Numerous previous studies have found that VRFs, such as hypertension, DM, and smoking, increase the risk of cognitive impairment (Kaddurah-Daouk et al., 2013; Ruiz-Canela et al., 2017). Our study was more rational and rigorous because FSRP was used to quantify multiple modifiable VRFs and to consider VCI as a “disease group.” Our results demonstrated that various VRFs accumulated aggravate cognitive impairment.

As the most common pathology type of VaD, subcortical vascular pathology always interrupts frontostriatal circuits and predominantly impairs cognitive domains of executive function, information processing, and attention (Stebbins et al., 2008). Other deficits in memory, verbal function, and praxis are much more variably affected in VaD. We found that patients with serum samples showed significantly poorer cognitive performance in terms of MoCA, RAVLT (immediate memory), RAVLT (delayed memory), and DSST in the higher risk groups. However, patients with CSF samples displayed significant worse cognitive performance in terms of MoCA, RAVLT (immediate memory), RAVLT (delayed memory), VFT, and DSST in the higher risk groups. Verbal function was thought to be associated with VCI (Jennifer et al., 2017), but we did not observe this association in the population who provided serum samples, probably because the number of samples was small. For the same reason, we observed no differences in attention between subgroups in both the serum and CSF groups. On the other hand, perhaps because of the early stage of VCI, attention damage was less obvious than the impairment of executive function. Memory impairment was evident in the higher risk groups, and we believe the cause might be related to the large age differences between subgroups. Age is one of the important VRFs that we must consider in our study. Additionally, we found that, in the correlation analysis between key metabolites and cognitive assessments, the correlation coefficient was relatively small, probably because the disease we studied belongs to the early stage of VCI, and the subjects studied were “relatively healthy.”

As VRFs accumulated, seven key differential metabolites in serum were upregulated (three of these metabolites were negatively correlated with cognitive impairment, and the other four metabolites showed no correlation with cognitive impairment), and one was downregulated (positively correlated with cognitive impairment). In CSF samples, three significantly differential metabolites were upregulated (negatively correlated with cognitive impairment). The effects of differential metabolites on cognitive impairment and vascular burden are consistent. Two metabolic pathways call our attention: caffeine metabolism (in serum and CSF) and TCA cycle metabolism (in serum). In our serum and CSF samples, regrettably, we found no common potential metabolites of VRF-associated cognitive impairment. The cause may be that brain metabolism has its own characteristics and the presence of the blood–brain barrier. Fortunately, we found intersections in the important metabolic pathways. In serum, four metabolites (1-methyluric acid, 3-tert-butyladipic acid, arachidonoyl PAF C-16, and Ile Cys Arg) were identified as potential biomarkers at the early stage of VCI. 1-Methyluric acid is the end product of the theophylline and caffeine metabolic pathway. 1-Methyluric acid was proven to be a predictor of moderately protect against peroxyl oxidation, was found to be a better radical scavenger than caffeine (León-Carmona and Galano, 2011) and can protect individuals against inflammation (Watanabe et al., 2008). Arachidonoyl PAF C-16 is a fatty acid, the product of acylation of lyso-PAF C-16 by a CoA-independent transacylase, and the most common precursor for the formation of PAF C-16 by the remodeling pathway. Ile Cys Arg is a small-molecule polypeptide whose function may be diverse. We can focus on the amino acids comprising this polypeptide. In CSF, three significantly changed metabolites [Asp His, 13-HOTrE(r), and 2,5-di-tert-Butylhydroquinone] were identified for the first time as potential biomarkers in stroke-free patients with VRFs. Asp His is a dipeptide comprising aspartate and histidine and is an incomplete breakdown product of protein digestion or protein catabolism. This dipeptide has not yet been identified in human tissues or biofluids; thus, it is classified as an “expected” metabolite. 2,5-Di-tert-Butylhydroquinone can play an antioxidant role by producing H_2_O_2_, a source of reactive oxygen species (Rhee, 2006; Bauman et al., 2018). 2,5-di-tert-Butylhydroquinone is the oxidation substrate used to measure the catalytic activity of copper (II) enzyme-like catalysts—that is, it is a useful tool to assess reactive oxygen species contributions (Jakubiak et al., 2006). 2,5-di-tert-Butylhydroquinone can partially inhibit sarco (endo) plasmic reticulum Ca^2+^ ATPase to suppress Ca^2+^-triggered arrhythmias (Bai et al., 2013). Compared with serum, CSF can better reflect the metabolism of the brain internal environment.

The exact pathological mechanism of cognitive impairment associated with VRFs remains unclear. It is generally believed that the VRF burden damages the neurovascular unit (Satizabal et al., 2016). Neurovascular unit damage can reduce cerebral vascular reactivity and the cerebral blood flow regulation function, can cause brain metabolism disorder, can destroy the blood–brain barrier, and can ultimately lead to brain atrophy, white matter degeneration, and cognitive impairment (Erdő et al., 2016; Kisler et al., 2017). Oxidative stress, abnormal energy metabolism, and the inflammatory response are involved in this pathological process (Chung et al., 2015; Raz et al., 2015).

Our findings are consistent with these effects. 1-Methyluric acid, arachidonoyl PAF C-16, and 2,5-di-tert-butylhydroquinone were shown to be antioxidants, and 1-methyluric acid also has an anti-inflammatory effect. The upregulation of 1-methyluric acid and 2,5-di-tert-butylhydroquinone may be a compensatory mechanism in response to possible early oxidative stress and inflammatory reactions that occur in the early stage of VCI subjects. The underlying meaning of caffeine metabolism changes should be similar. Changes in the TCA cycle indicated the state of energy metabolism compensation in the organism. Once the compensation is lost, the disease will be aggravated; at the same time, the energy supply and oxygen supply will be insufficient (Dichgans and Leys, 2017), eventually leading to changes in the intracellular environment and even cell necrosis. Previous studies have found that serum TCA cycle, lipid metabolism, amino acid metabolism, and purine metabolism in subcortical infarction patients with VCI showed substantial changes compared with those in the normal control group and subcortical infarction without the VCI group (Zhou and Bi, 2014). These findings were also consistent with ours. The basis to change in the levels of metabolites may be that the accumulation of VRFs damages the vascular endothelium, resulting in oxidative stress and energy metabolism disorders.

We excluded patients with obesity because they have a higher risk of cognitive impairment than non-obese patients (O’Brien et al., 2017). Obesity has been shown to be associated with AD-like neuropathology (β-amyloid, tau) in the hippocampus and a decrease in the hippocampal volume (Jagust et al., 2005; Mrak, 2009). Better education is considered to be an important factor in higher cognitive reserve (Stern et al., 1994, 1999), which is considered to be one of the crucial factors affecting cognitive function (Stern et al., 1999). Therefore, when comparing the cognitive scores in subgroups, we used the degree of education as a covariate. We chose patients undergoing elective surgery to collect CSF samples, such as implant removal of the low extremity and inguinal hernia. Patients undergoing such surgeries were selected to exclude the possible effects of traumatic stress and pain on metabolism and cognitive assessment. The cognitive assessments were performed before surgery. All the biological samples were collected at 8:00–10:00 AM and under fasting conditions to ensure that the detected metabolism occurred at the same time. We tried our best to be rigorous, which represents one of the highlights of the study. Although we eliminated patients with definite dementia, we could not exclude early AD patients due to the lack of further neuroimaging, neuropathology, and cerebrospinal fluid biomarker detection, which would be an important weakness in our study.

In summary, our work demonstrated significantly altered cognitive function in stroke-free patients with different FSRP scores. Through UPLC-MS/MS-based metabolomics technology, we provided nontargeted metabolic profiles and key metabolic pathways for VRF-related cognitive impairment. The findings in the current study could be further validated and investigated in several ways. First, a larger number of serum, CSF, and other biologic samples are needed for population-based validation; second, a semiquantitative method was used in our study. LCMS/MS is characterized by high efficiency, high sensitivity, and fast analysis. The advantages of UPLC-MS/MS are more prominent. For future clinical applications, absolute quantitative analysis is recommended for stable and reliable biomarker detection and monitoring. Finally, the seven metabolites and two metabolic pathways need to be further investigated.

## Data Availability Statement

The datasets analyzed in this article are not publicly available. Requests to access the datasets should be directed to SP, cissyi@hotmail.com.

## Ethics Statement

The studies involving human participants were reviewed and approved by the medical ethics committee of Zhongnan Hospital, Wuhan, China (clinical research registration number 2016007). The patients/participants provided their written informed consent to participate in this study.

## Author Contributions

SP and YS completed the experiment together. MW assisted the data analysis. SP wrote the manuscript. JZ helped revise the text to the final form.

## Conflict of Interest

The authors declare that the research was conducted in the absence of any commercial or financial relationships that could be construed as a potential conflict of interest.

## Footnotes

^1^https://www.framinghamheartstudy.org/fhs-risk-functions/stroke/

^2^http://www.genome.jp/kegg/

^3^http://www.metaboanalyst.ca/

## Supplementary Material

FIGURE S1 Principal component analysis (PCA) score plot derived from the liquid chromatography-tandem mass spectrometry (LC-MS) metabolite profiles of serum samples. Low-risk group, middle-risk group, high-risk group and quality control (QC) group.

FIGURE S2 PCA score plot derived from the LC-MS metabolite profiles of CSF samples. Low-risk group, middle-risk group, high-risk group and QC group.

FIGURE S3 Orthogonal projections to latent structures-discriminate analysis (OPLS-DA) score plots and corresponding validation plots of OPLS-DA from the LC-MS metabolite profiles in the serum of the high-risk group and low-risk group.

FIGURE S4 OPLS-DA score plots and corresponding validation plots of OPLS-DA from the LC-MS metabolite profiles in the serum of the middle-risk group and low-risk group.

FIGURE S5 OPLS-DA score plots and corresponding validation plots of OPLS-DA from the LC-MS metabolite profiles in the CSF of the high-risk group and low-risk group.

FIGURE S6 OPLS-DA score plots and corresponding validation plots of OPLS-DA from the LC-MS metabolite profiles in the CSF of the middle-risk group and low-risk group.

FIGURE S7 Caffeine metabolism.
