# Supplementary material for: Serum and CSF Metabolites in Stroke-Free Patients Are Associated With Vascular Risk Factors and Cognitive Performance
Source: Front Aging Neurosci. 2020 Jul 22;12:193. doi: 10.3389/fnagi.2020.00193 (PMC7387721; doi:10.3389/fnagi.2020.00193)
Supplement: FIGURE S1 — Principal component analysis (PCA) score plot derived from the liquid chromatography-tandem mass spectrometry (LC-MS) metabolite profiles of serum samples. Low-risk group, middle-risk group, high-risk group and quality control (QC) group. [file Data_Sheet_1.docx]

**Supplementary Figures**

Supplementary Figure S1

Serum, ESI+

Serum, ESI-


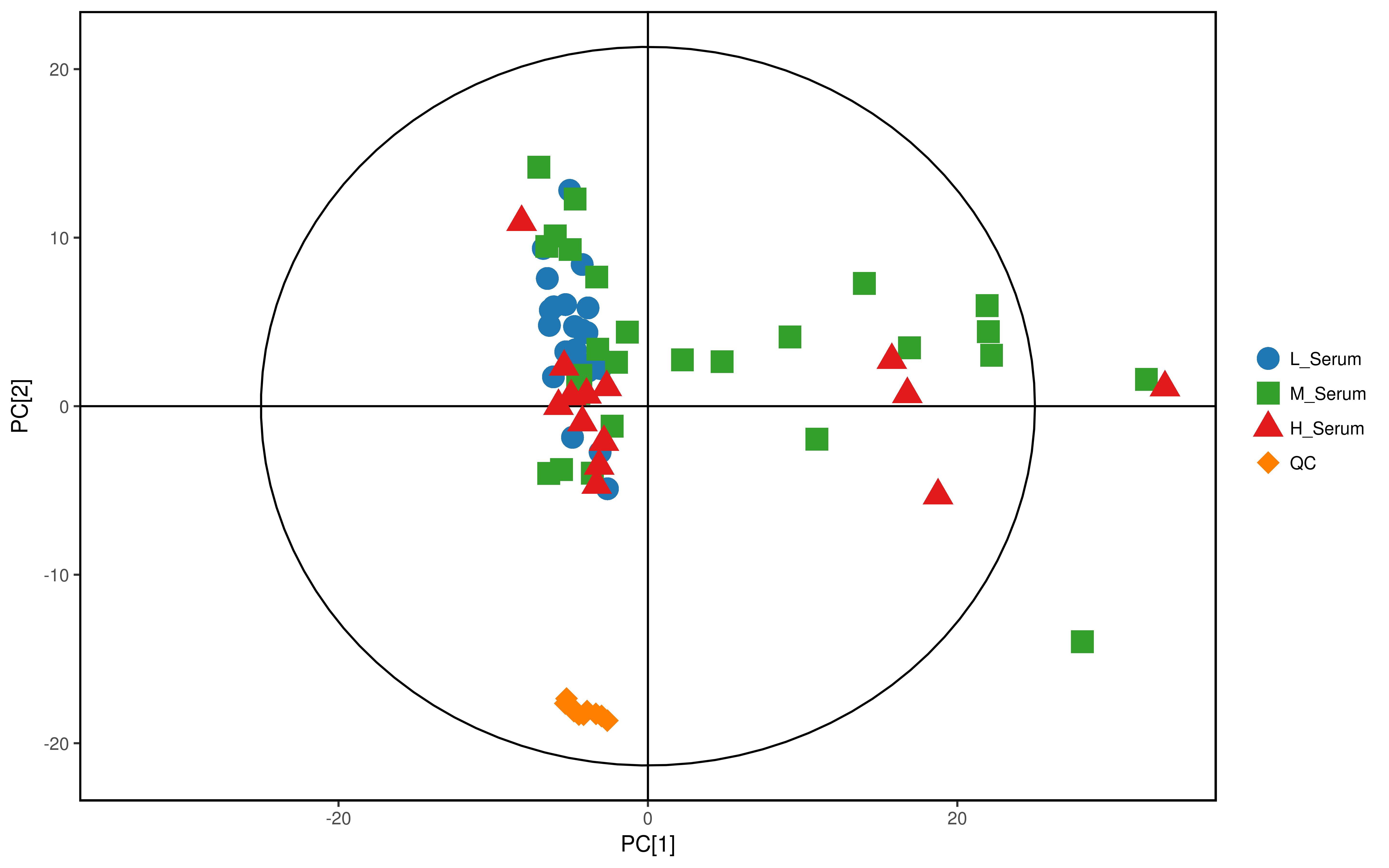

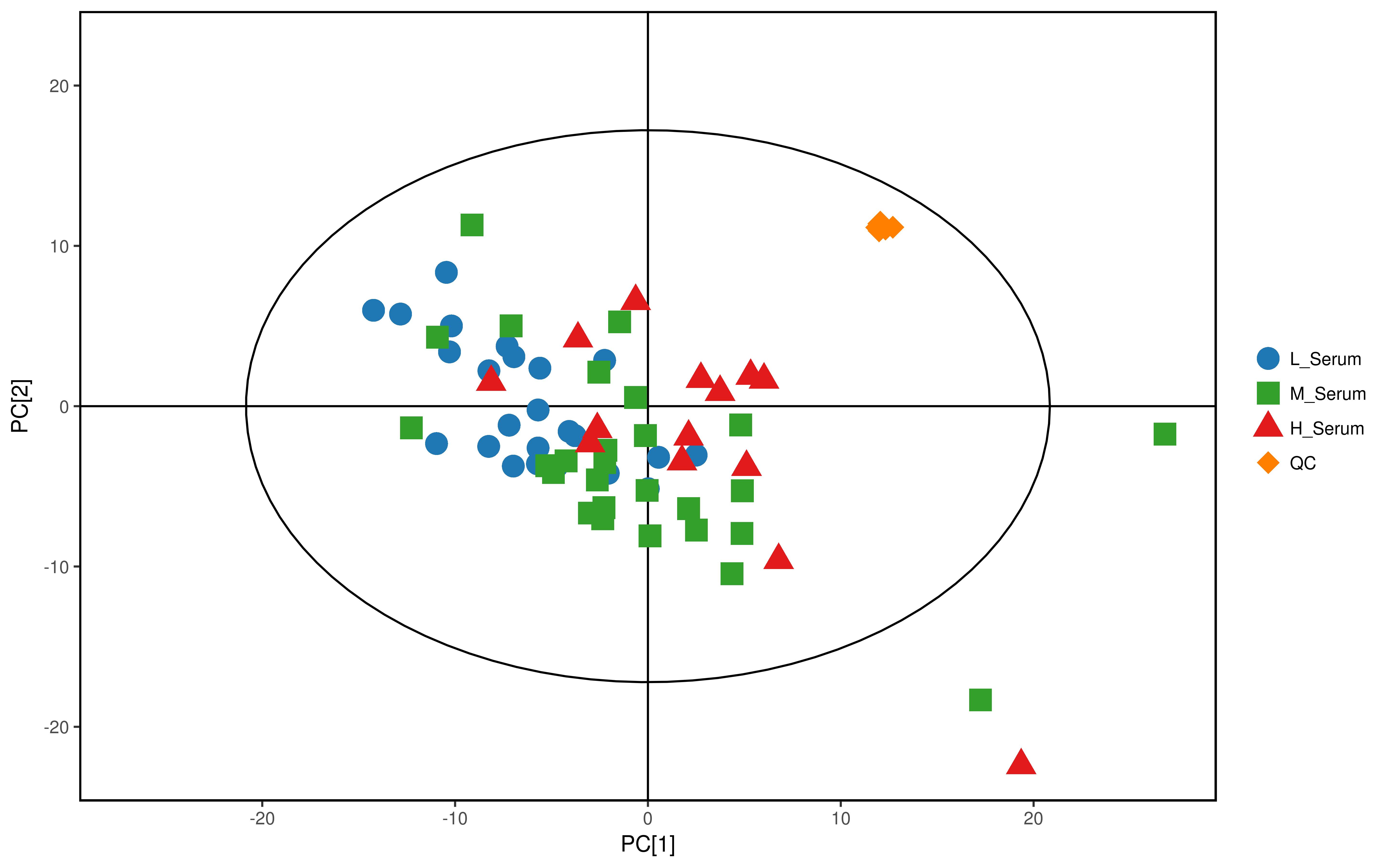


**Supplementary Figure S1**. PCA score plot derived from the LC-MS metabolite profiles of serum samples. Low-risk group, middle-risk group, high-risk group and QC group.

Supplementary Figure S2

CSF, ESI-

CSF, ESI+


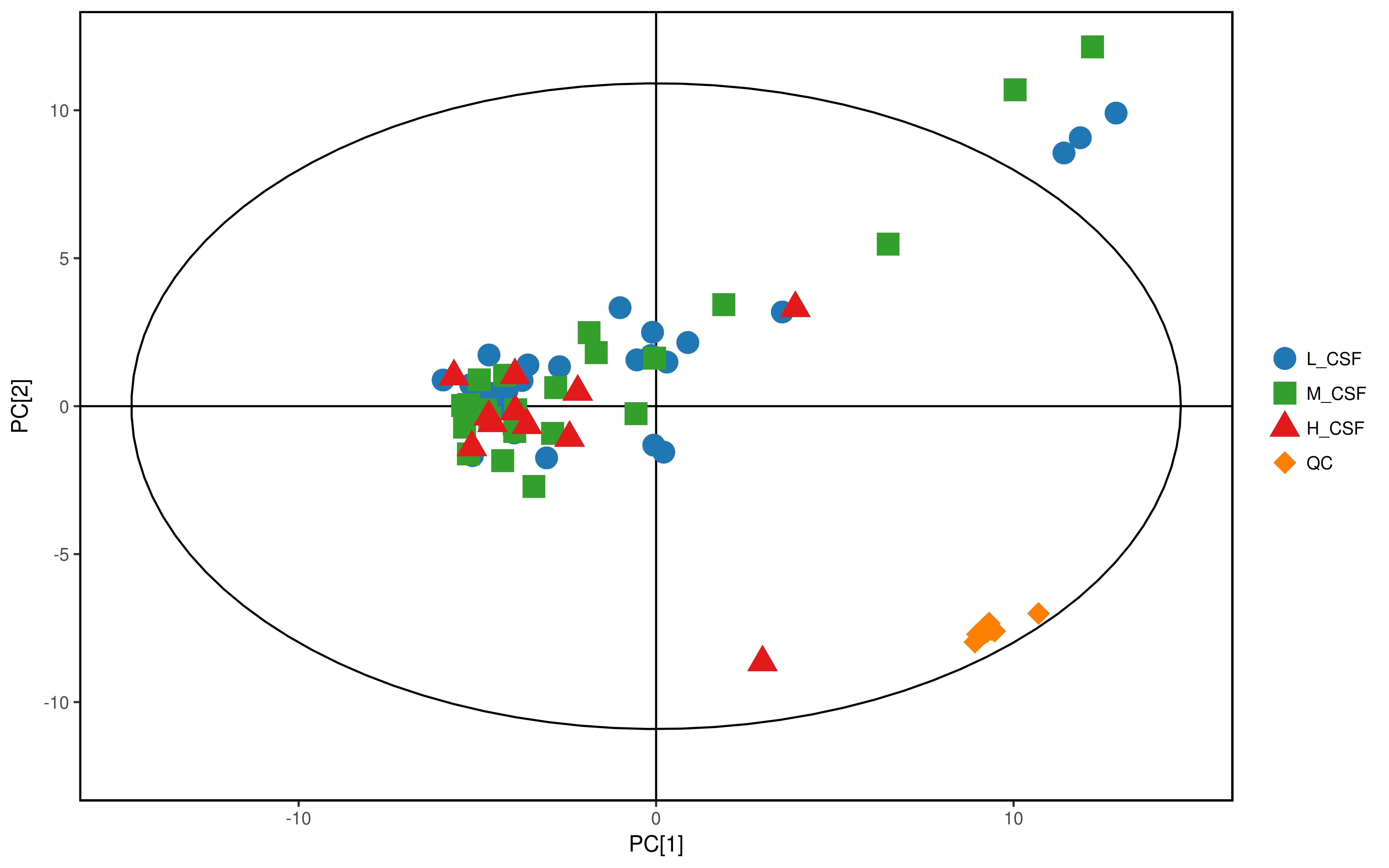

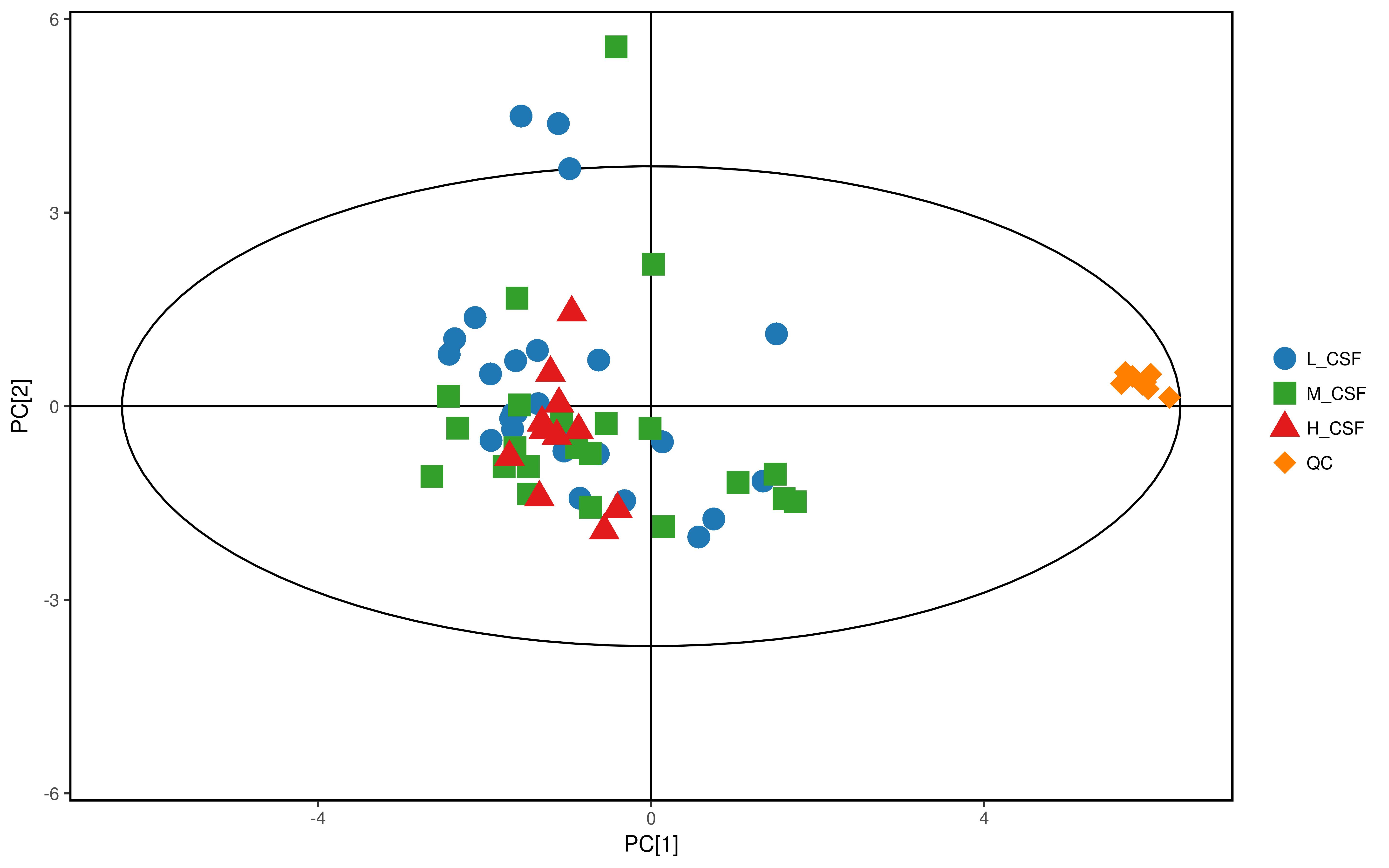


**Supplementary Figure S2**. PCA score plot derived from the LC-MS metabolite profiles of CSF samples. Low-risk group, middle-risk group, high-risk group and QC group.

Supplementary Figure S3

Serum


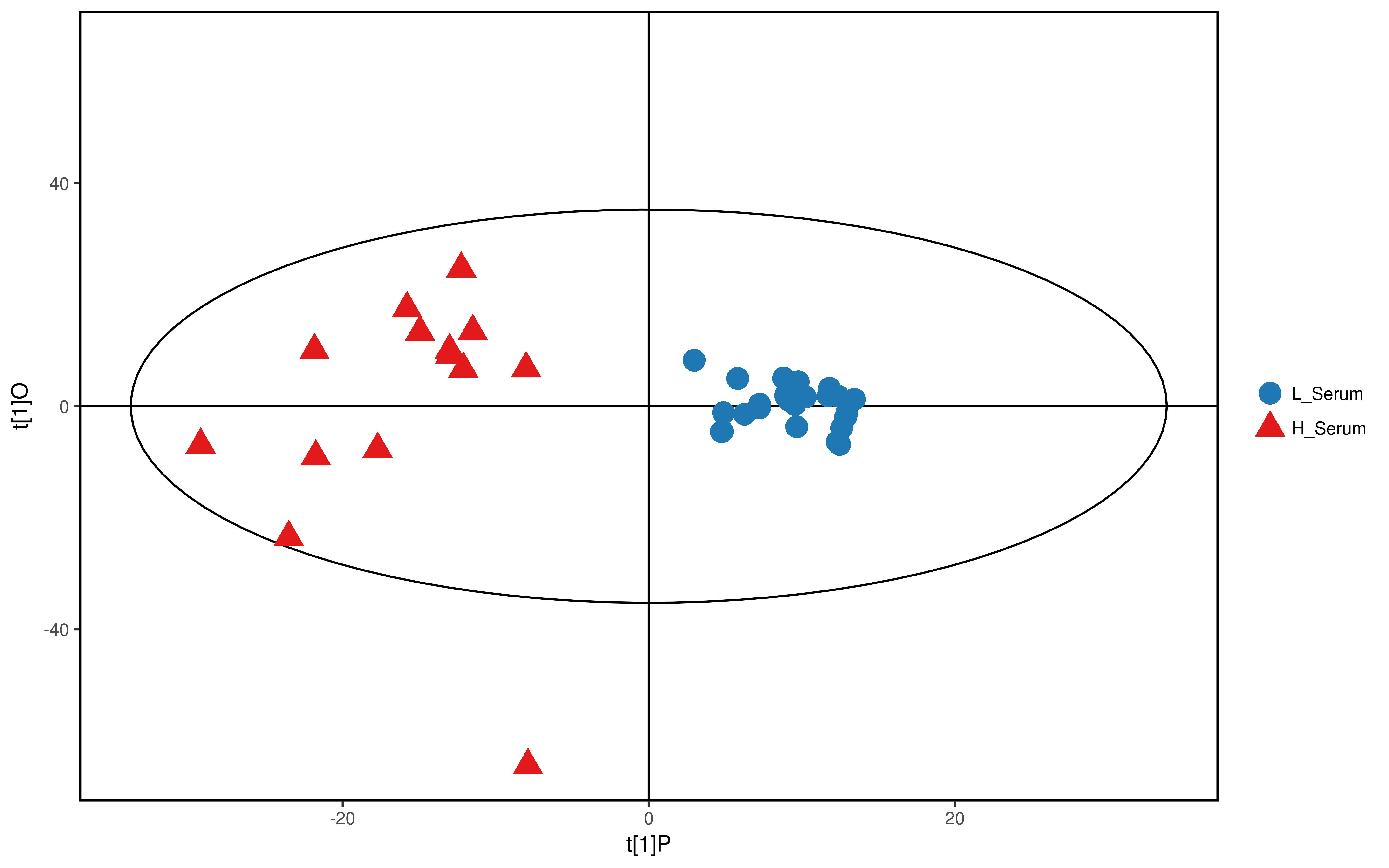

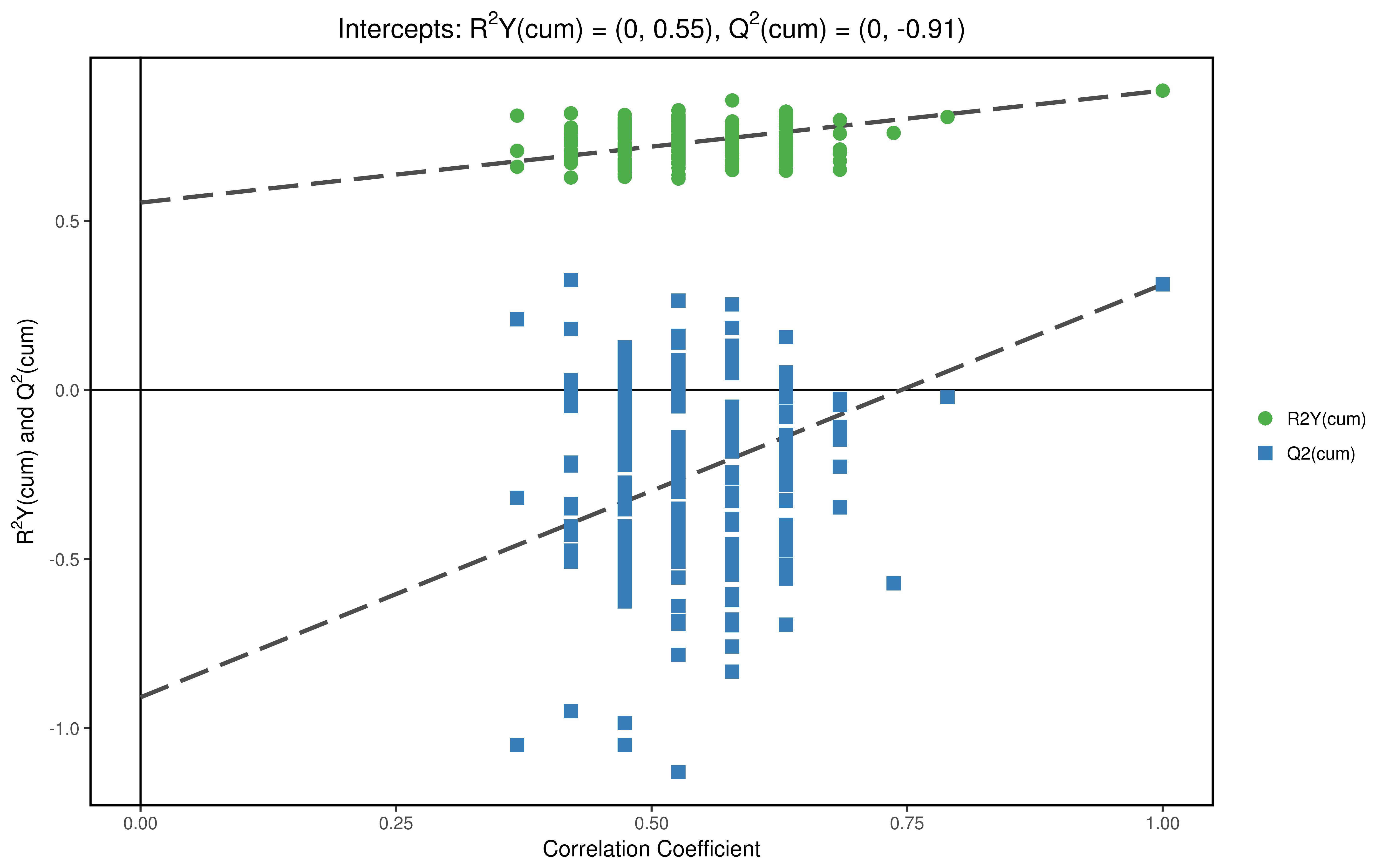


ESI+


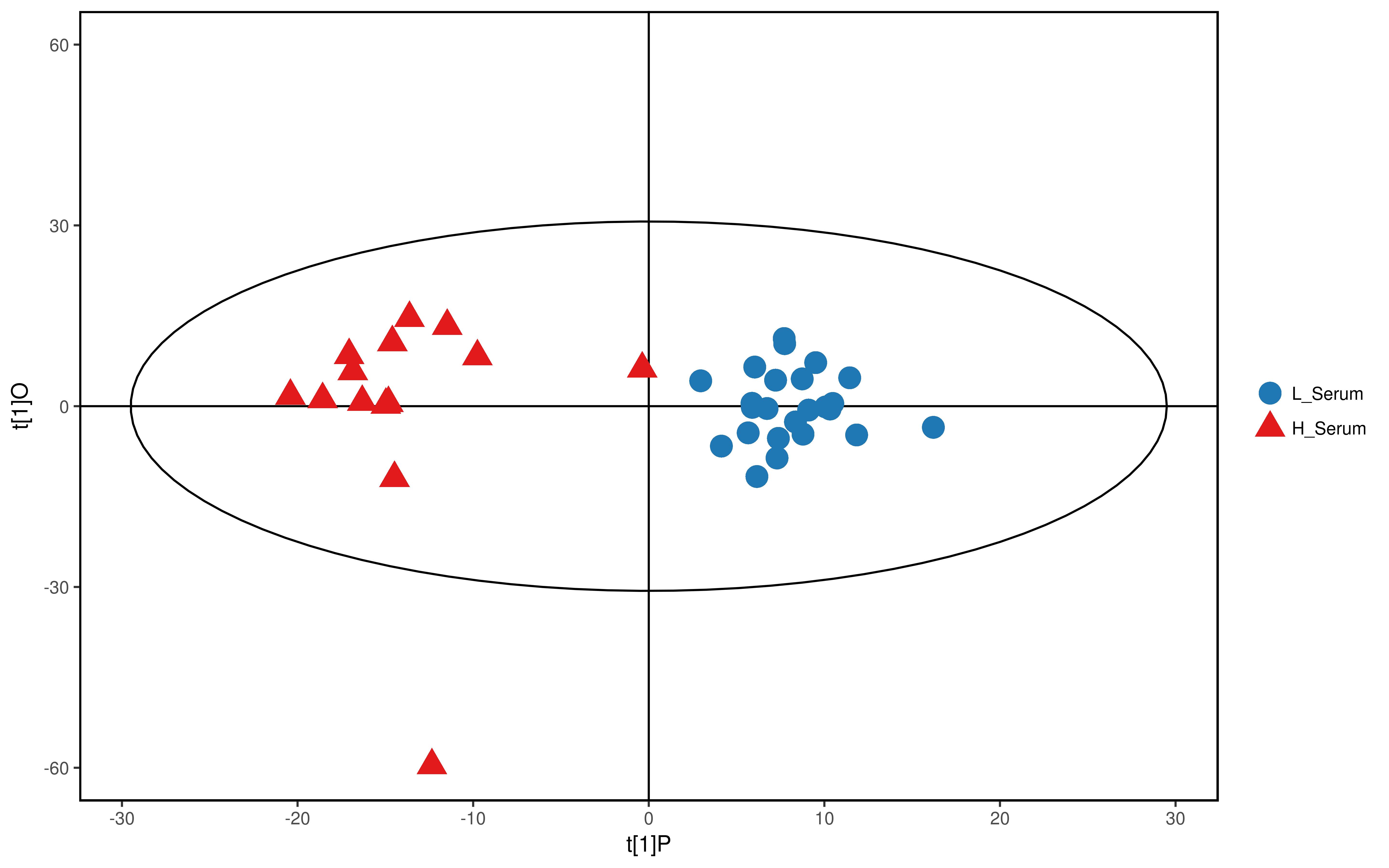

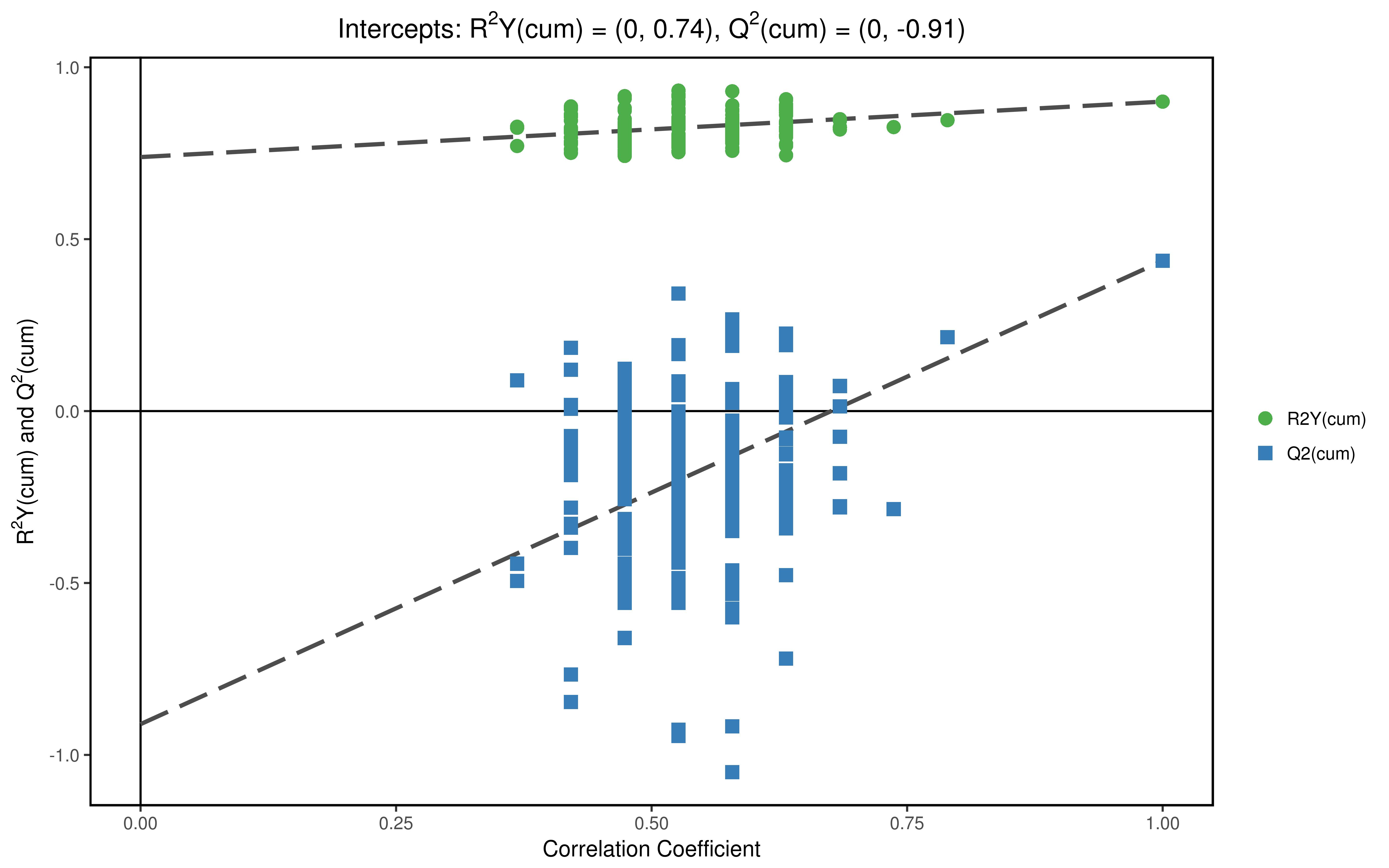


ESI-

**Supplementary Figure S3**. OPLS-DA score plots and corresponding validation plots of OPLS-DA from the LC-MS metabolite profiles in the serum of the high-risk group and low-risk group.

Supplementary Figure S4

Serum


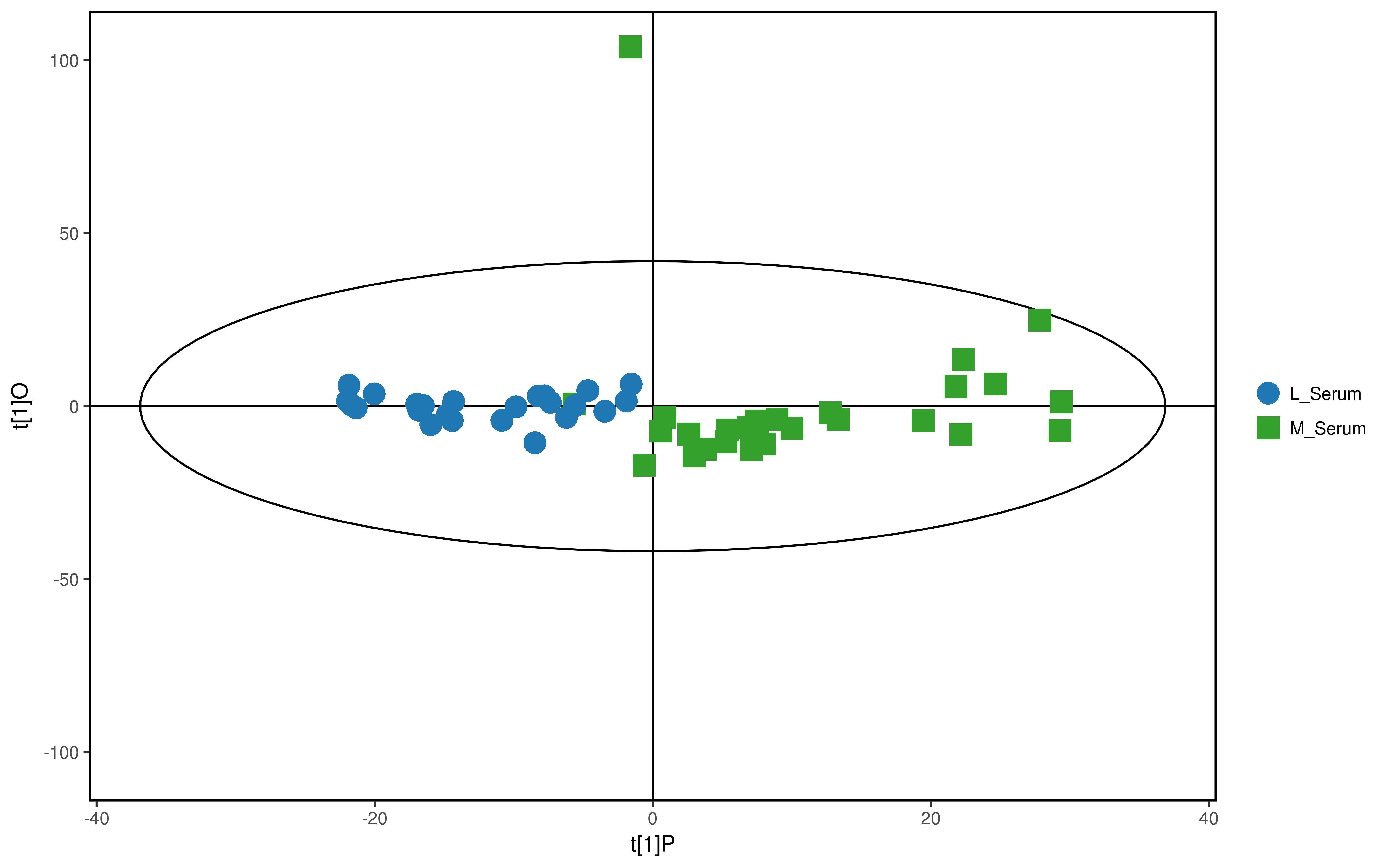

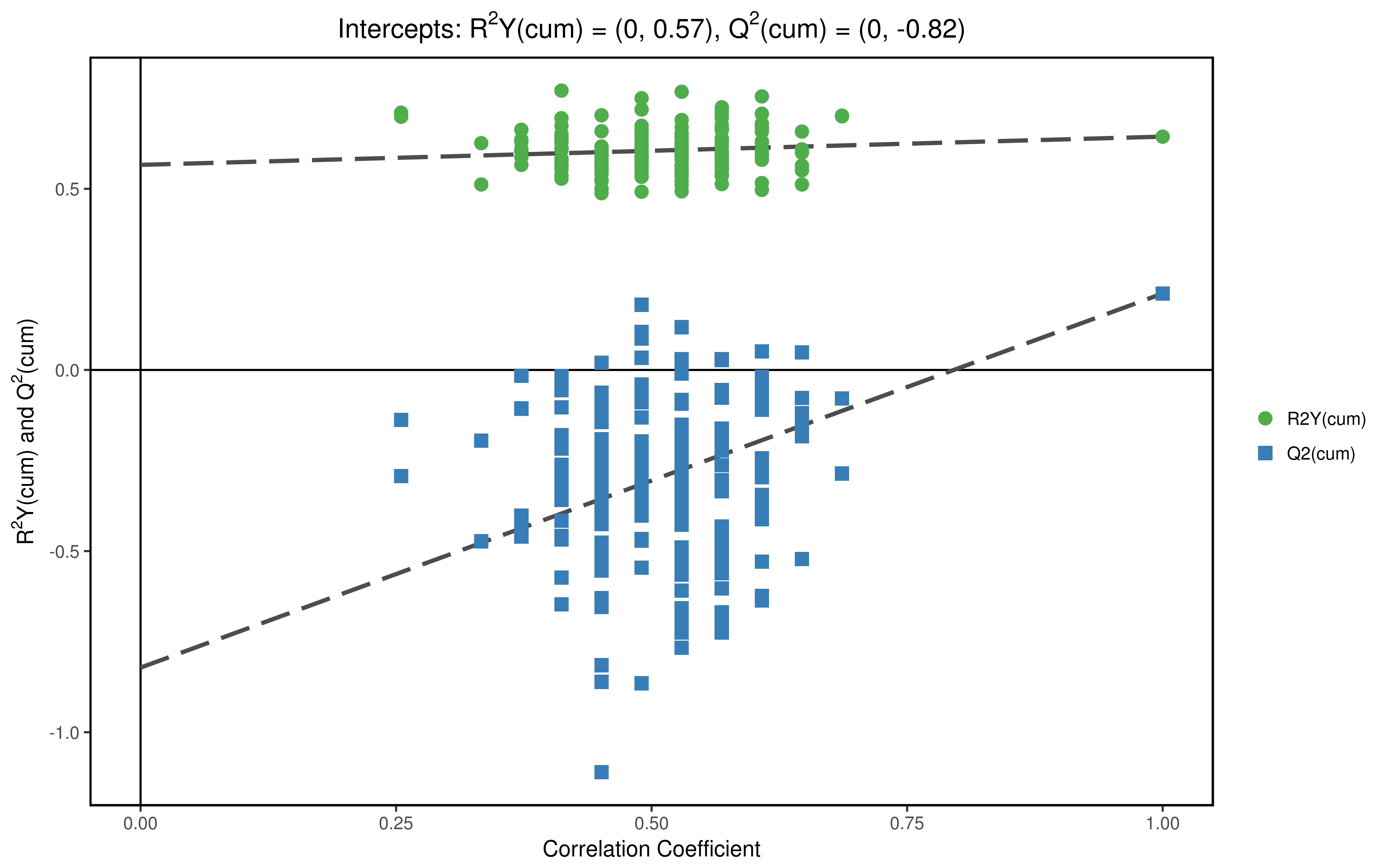


ESI-

ESI+


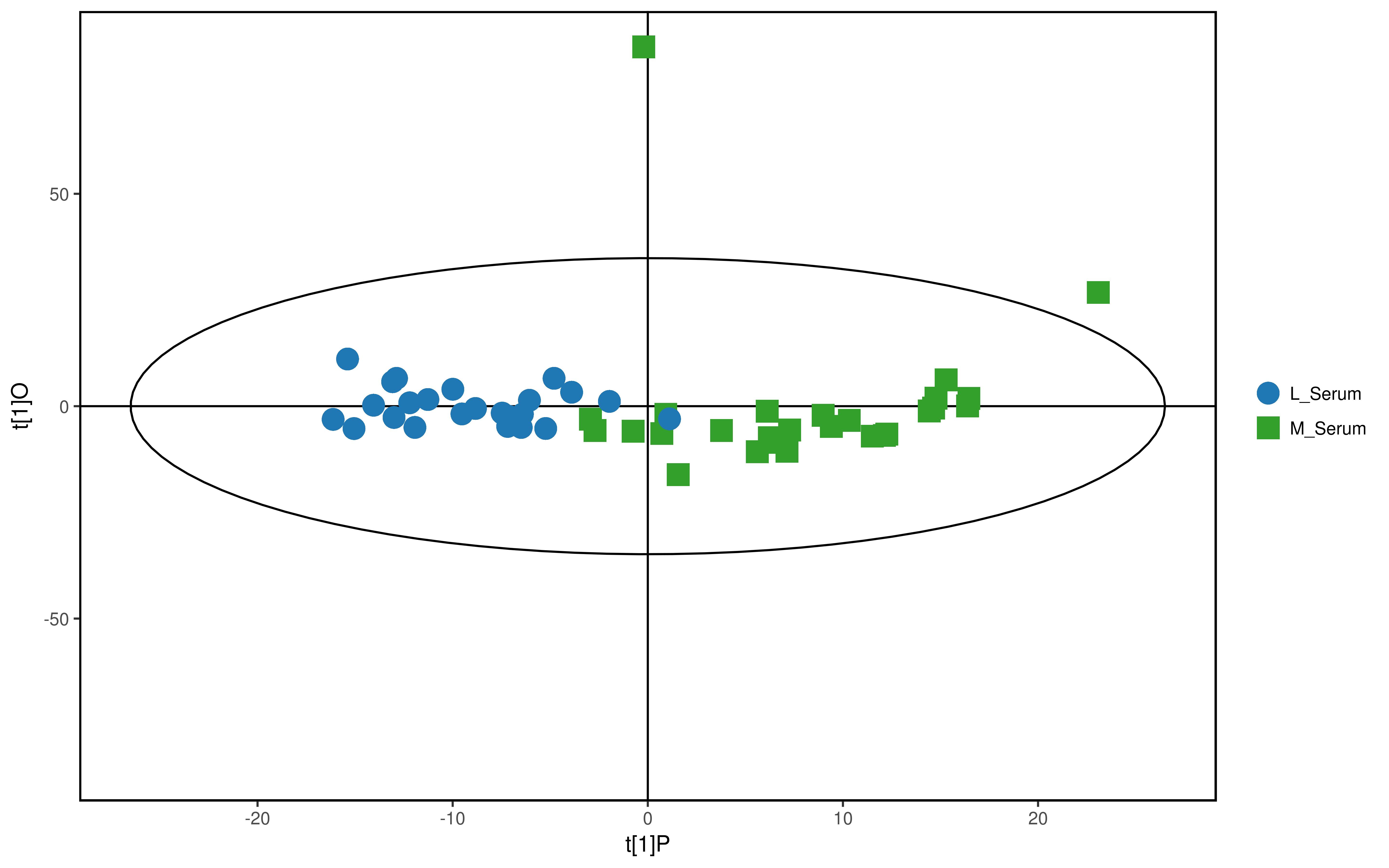

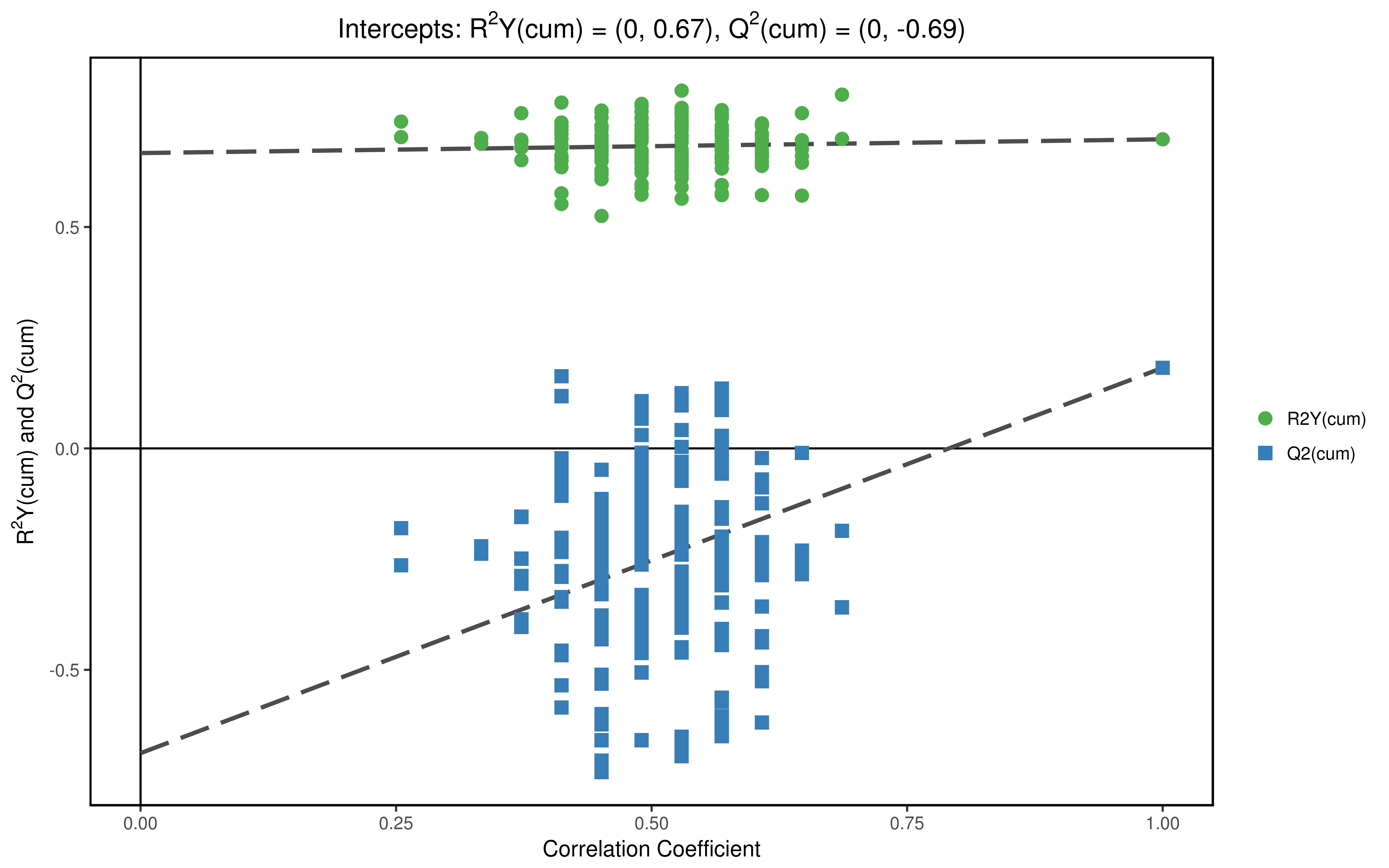


**Supplementary Figure S4**. OPLS-DA score plots and corresponding validation plots of OPLS-DA from the LC-MS metabolite profiles in the serum of the middle-risk group and low-risk group.

Supplementary Figure S5

CSF


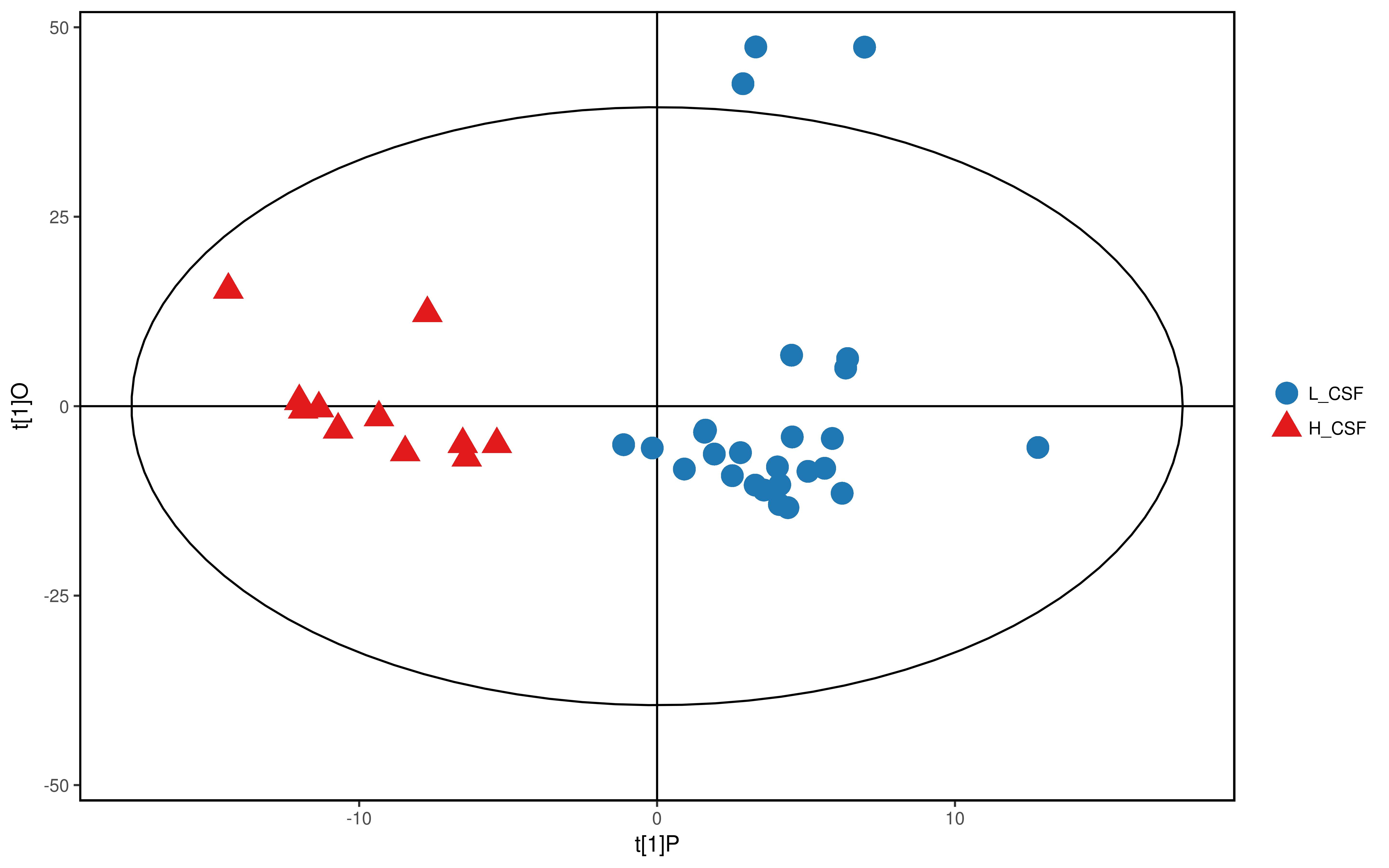

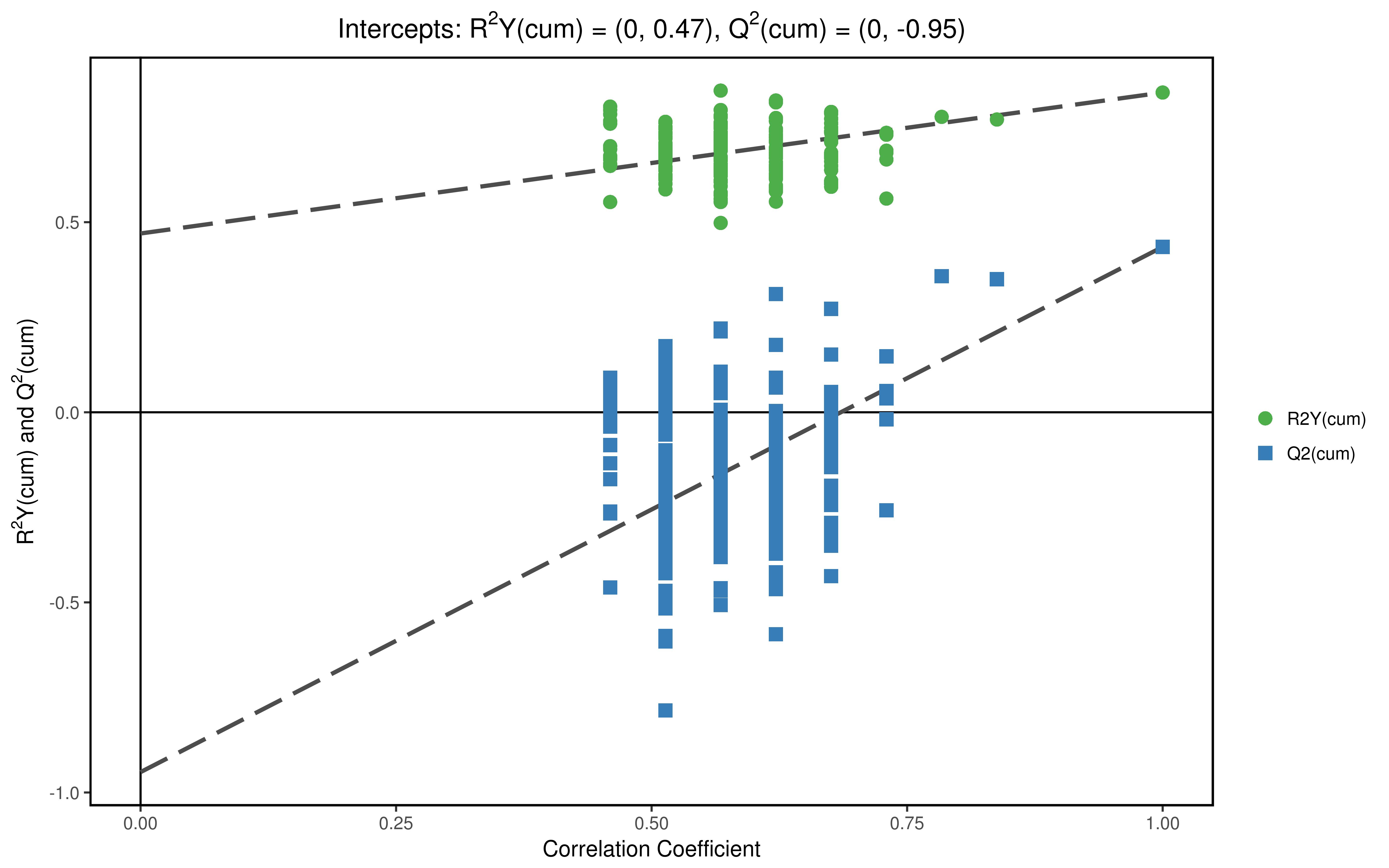


ESI+


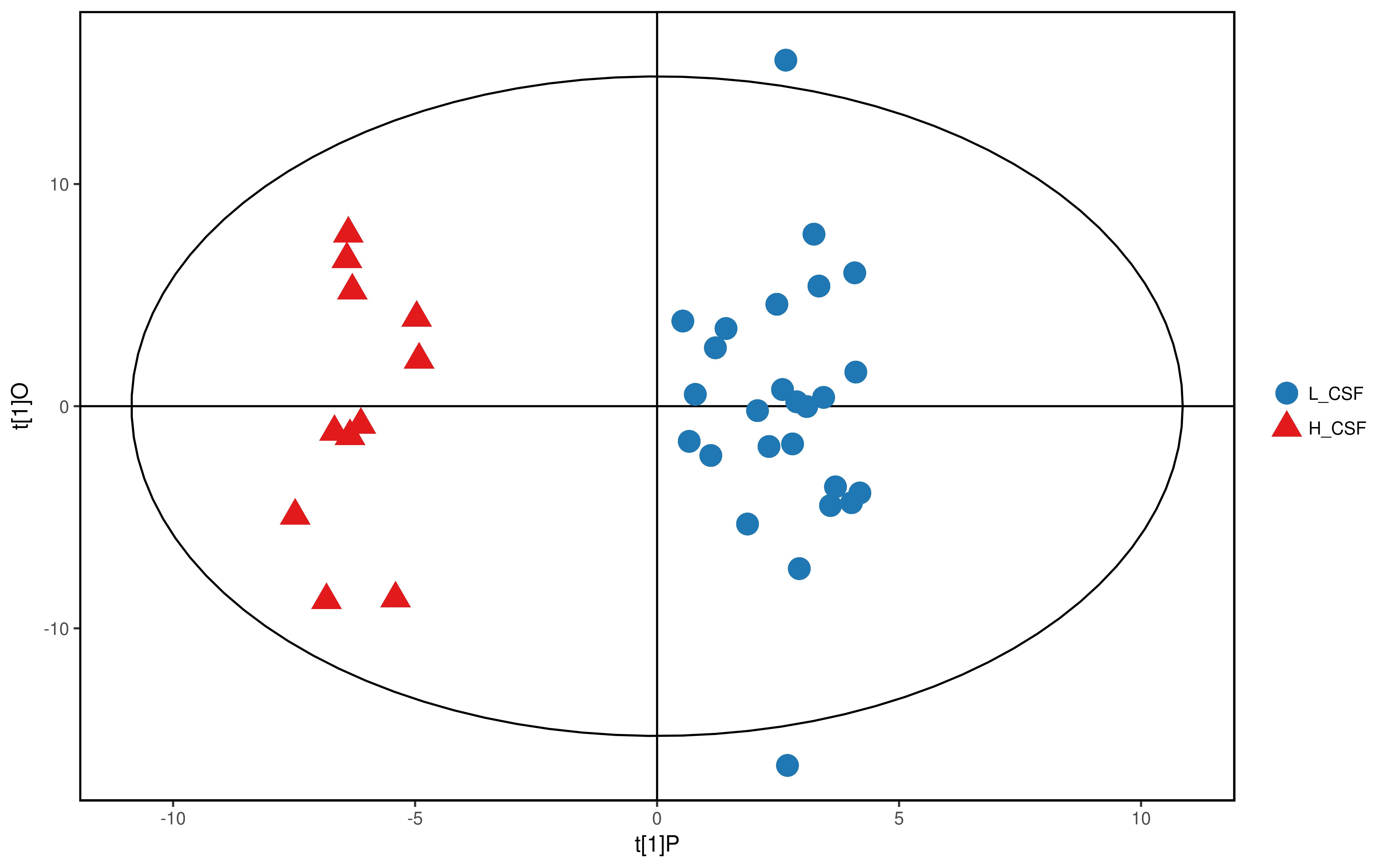

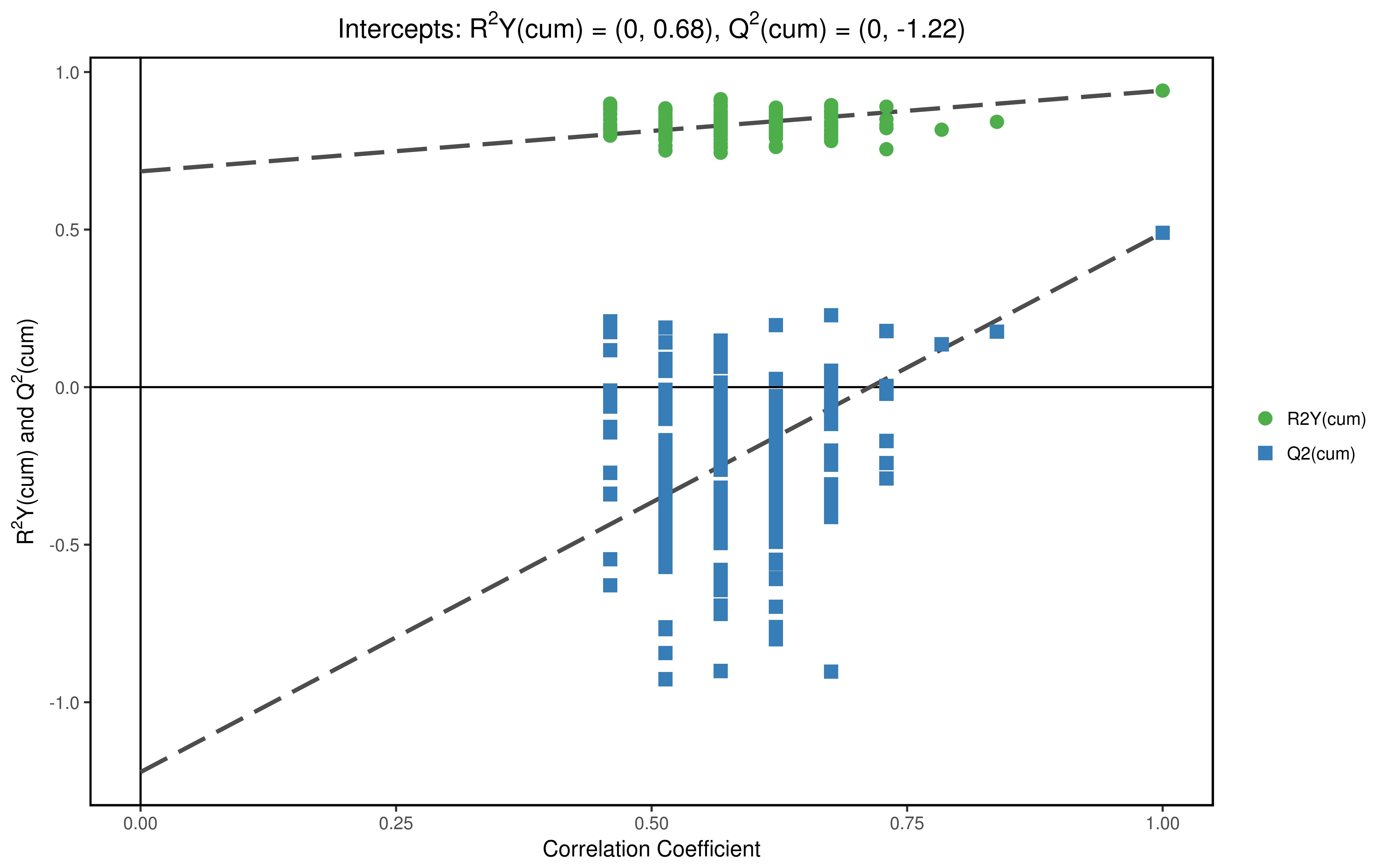


ESI-

**Supplementary Figure S5**. OPLS-DA score plots and corresponding validation plots of OPLS-DA from the LC-MS metabolite profiles in the CSF of the high-risk group and low-risk group.

Supplementary Figure S6

CSF


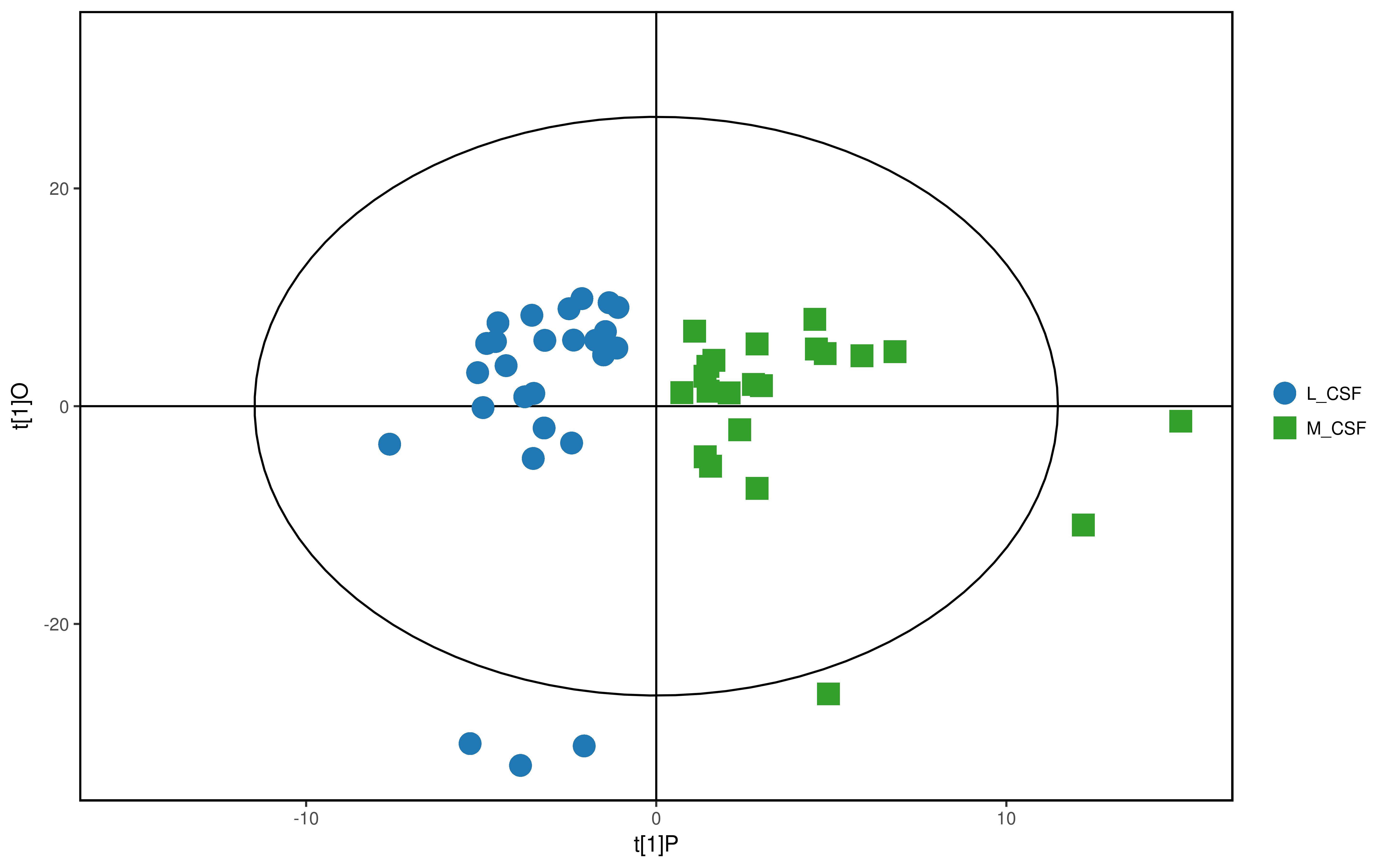

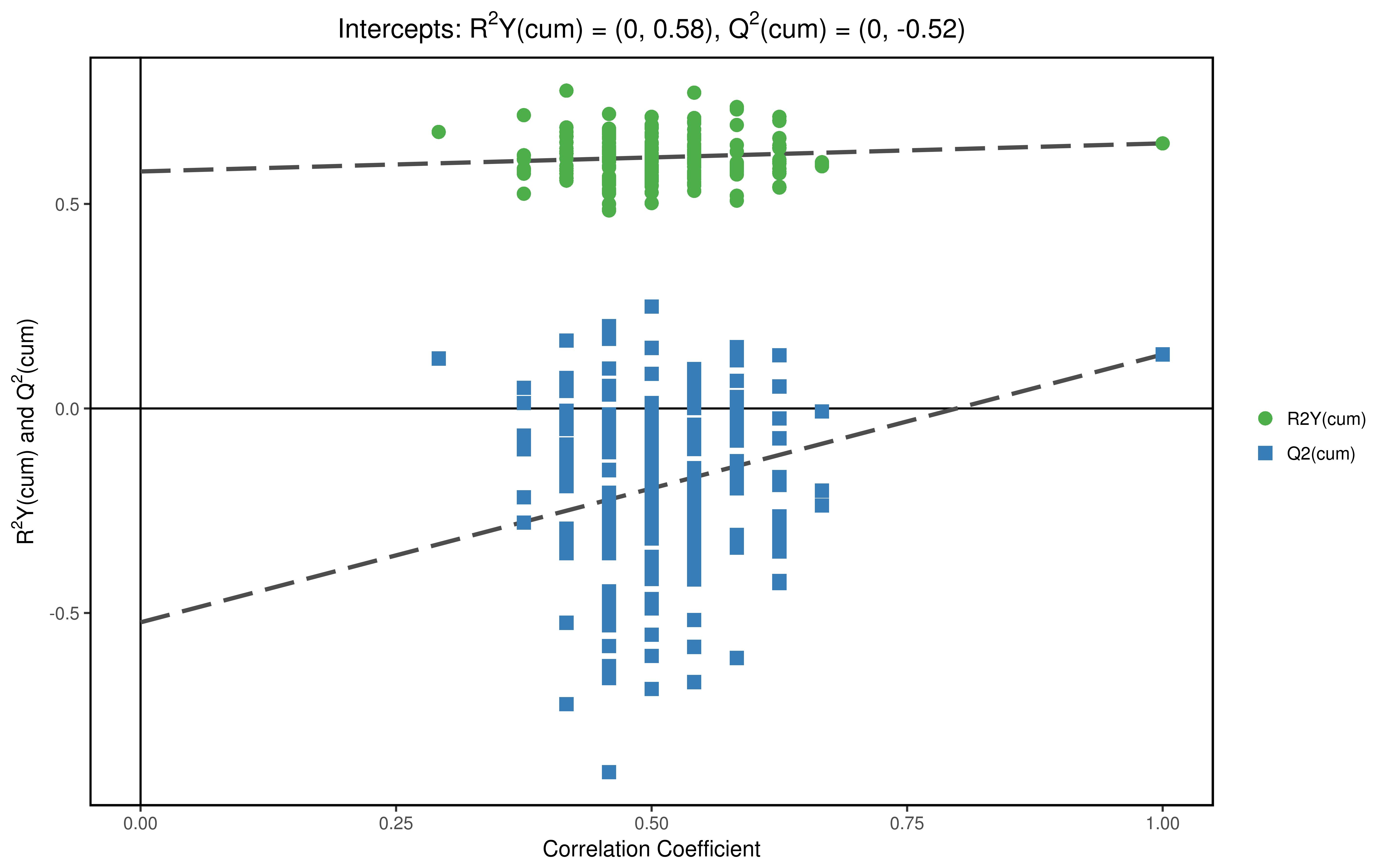


ESI+


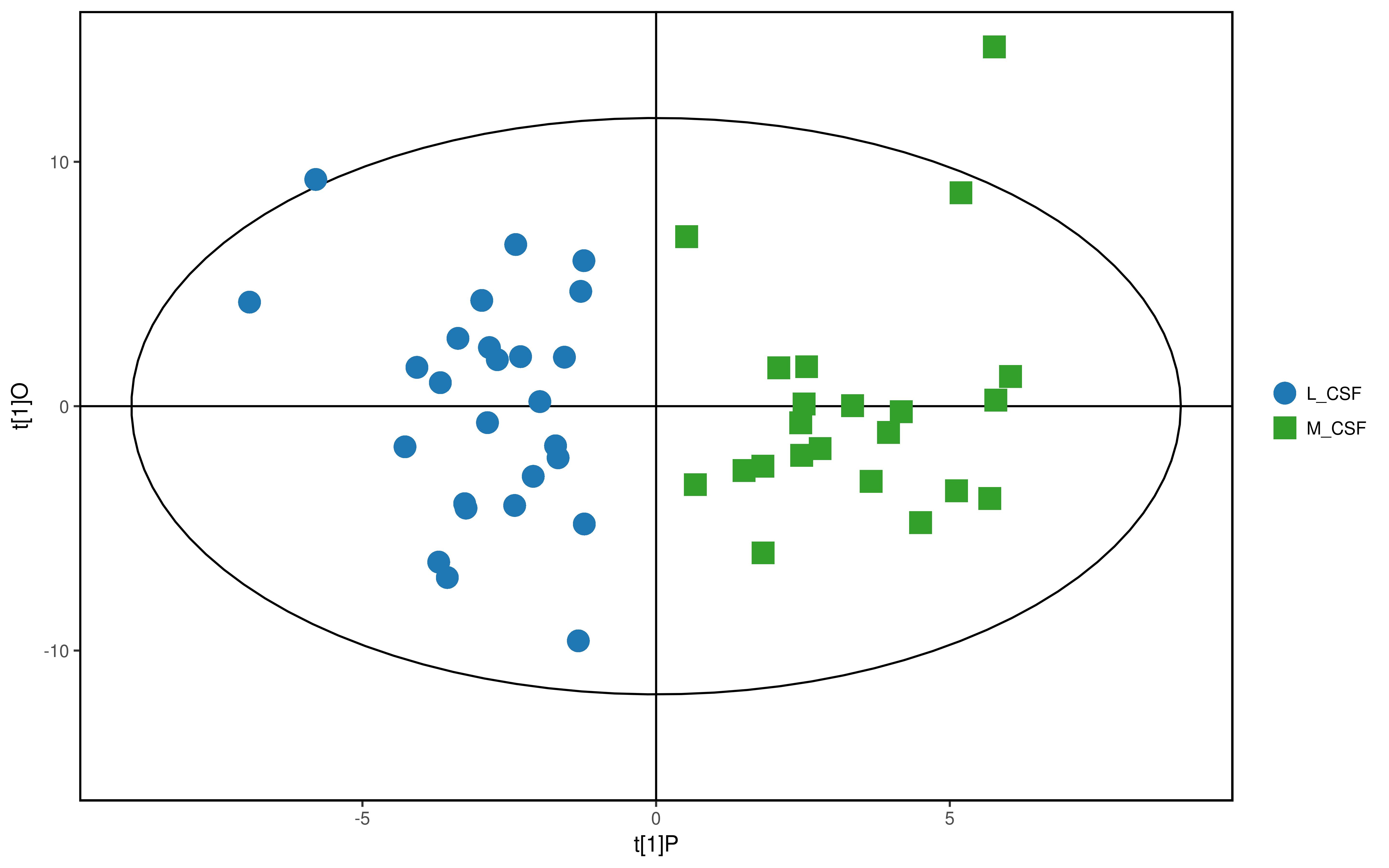

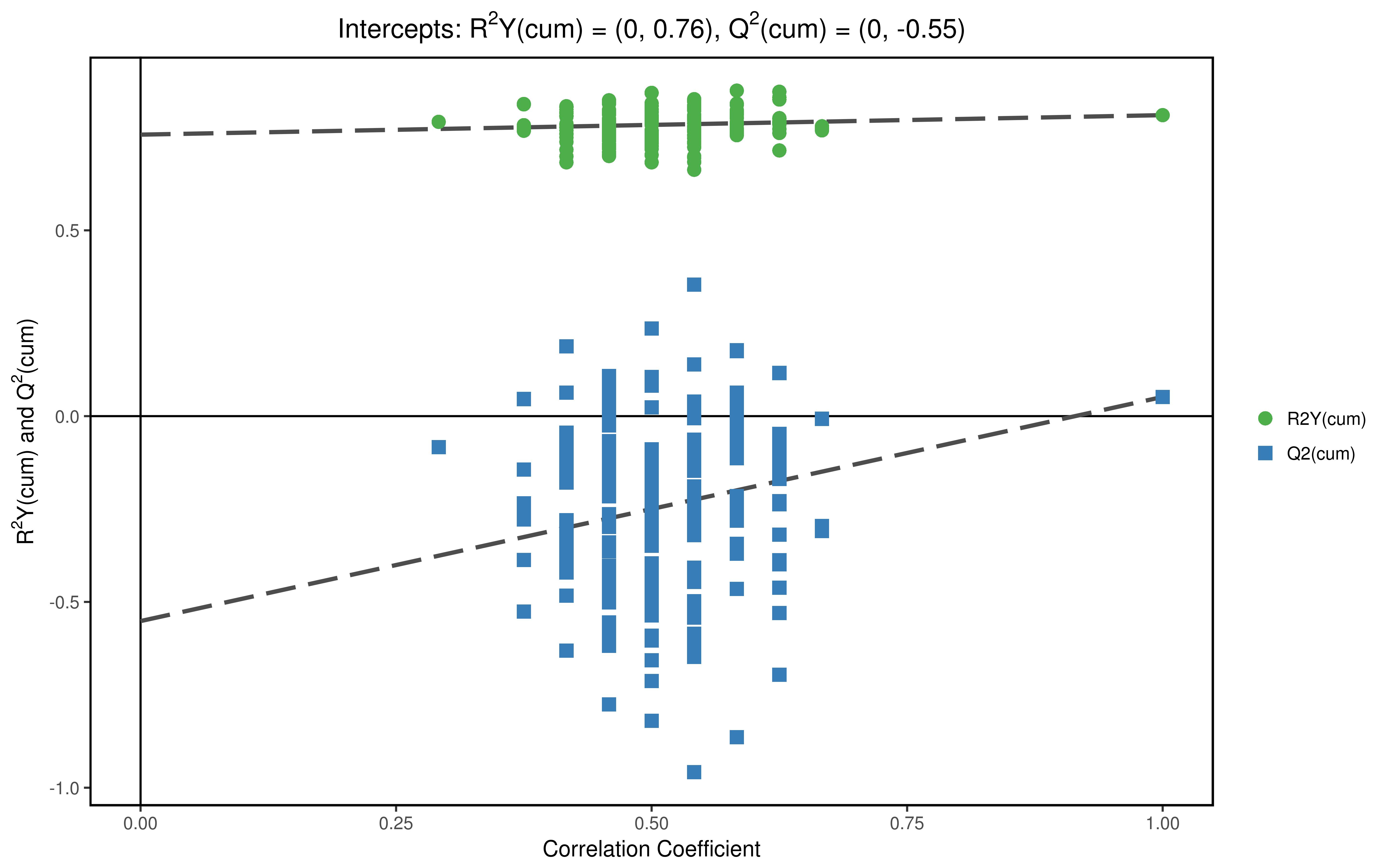


ESI-

**Supplementary Figure S6**. OPLS-DA score plots and corresponding validation plots of OPLS-DA from the LC-MS metabolite profiles in the CSF of the middle-risk group and low-risk group.

Supplementary Figure S7


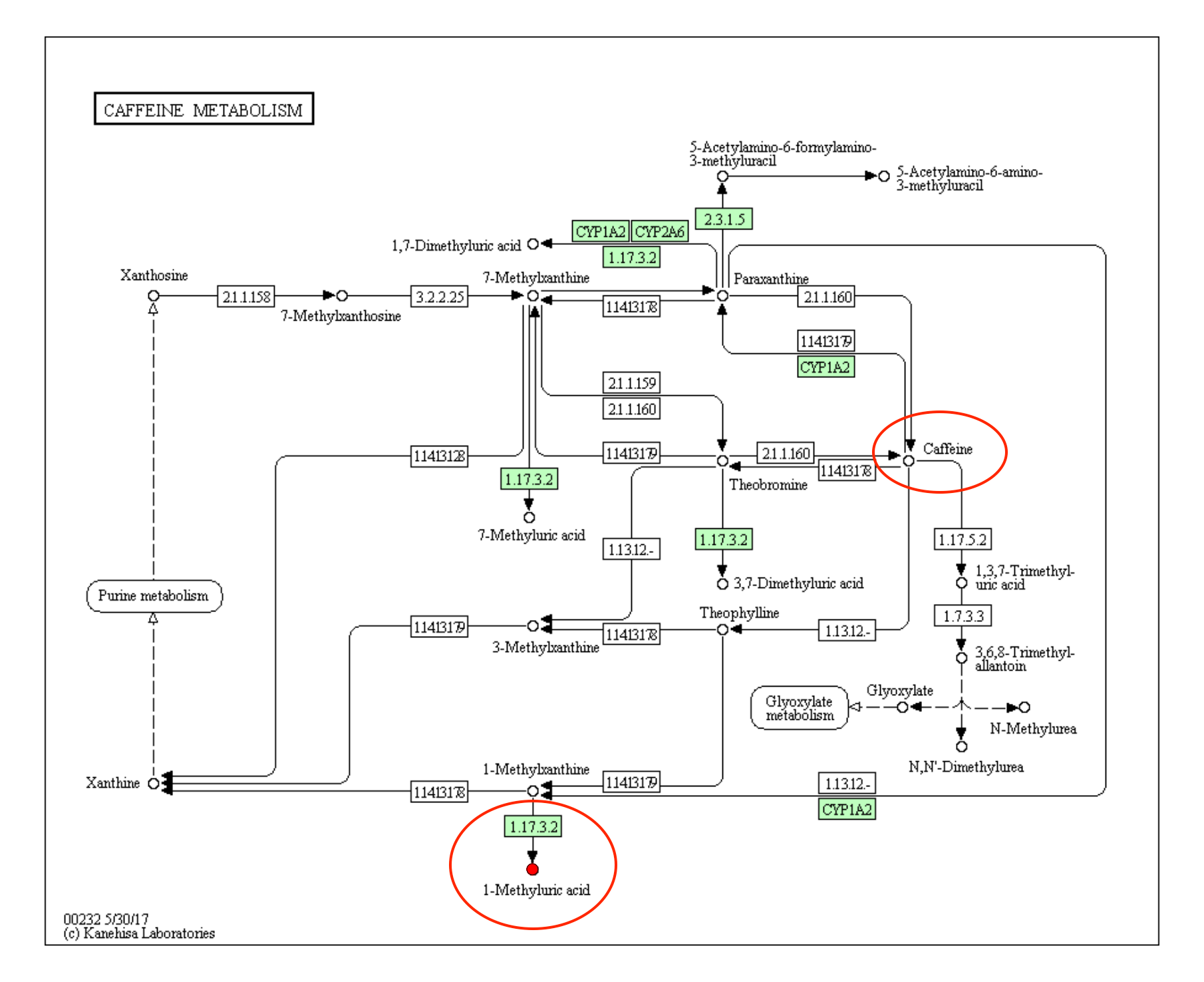


**Supplementary Figure S7**. Caffeine metabolism
